# Knowledge of the perturbation design is essential for accurate gene regulatory network inference

**DOI:** 10.1038/s41598-022-19005-x

**Published:** 2022-10-03

**Authors:** Deniz Seçilmiş, Thomas Hillerton, Andreas Tjärnberg, Sven Nelander, Torbjörn E. M. Nordling, Erik L. L. Sonnhammer

**Affiliations:** 1 Department of Biochemistry and Biophysics, Stockholm University, Science for Life Laboratory, Box 1031, 17121 Solna, Sweden; 2 Center for Developmental Genetics, New York University, New York, USA; 3 Department of Immunology, Genetics and Pathology and Science for Life Laboratory, Uppsala University, 75185 Uppsala, Sweden; 4 Department of Mechanical Engineering, National Cheng Kung University, Tainan, 701 Taiwan, ROC; 5 Department of Applied Physics and Electronics, Umeå University, 90187 Umeå, Sweden

**Keywords:** Regulatory networks, Gene regulatory networks

## Abstract

The gene regulatory network (GRN) of a cell executes genetic programs in response to environmental and internal cues. Two distinct classes of methods are used to infer regulatory interactions from gene expression: those that only use observed changes in gene expression, and those that use both the observed changes and the perturbation design, i.e. the targets used to cause the changes in gene expression. Considering that the GRN by definition converts input cues to changes in gene expression, it may be conjectured that the latter methods would yield more accurate inferences but this has not previously been investigated. To address this question, we evaluated a number of popular GRN inference methods that either use the perturbation design or not. For the evaluation we used targeted perturbation knockdown gene expression datasets with varying noise levels generated by two different packages, GeneNetWeaver and GeneSpider. The accuracy was evaluated on each dataset using a variety of measures. The results show that on all datasets, methods using the perturbation design matrix consistently and significantly outperform methods not using it. This was also found to be the case on a smaller experimental dataset from *E. coli*. Targeted gene perturbations combined with inference methods that use the perturbation design are indispensable for accurate GRN inference.

## Introduction

Accurate identification of gene interactions that regulate biochemical mechanisms in a living organism can help identify physiological and pathological mechanisms and enable researchers to e.g. understand the cause of genetic diseases. Prediction of these gene regulatory interactions can be performed from gene expression data via gene regulatory network inference methods which differ among each other in terms of their mathematical models. The accuracy of one inference method may exhibit fluctuations based on the properties of the dataset, e.g. noise levels^1,2^. Several benchmark studies have been published from the Dialogue on Reverse Engineering Assessment and Methods (DREAM) network inference challenges^3–5^, where different sources of networks and data were used in each challenge, and performance comparisons were made for different methods. These benchmarks, especially the fifth round of DREAM^5^ provided a broad selection of GRN inference methods, however, they did not assess the performances of these methods at different data properties such as noise levels. A benchmark of ten GRN inference methods by Bellot et al.^6^ includes an analysis of the impact of noise, but did not consider link direction, and all measured accuracies are very low, which reduces its usefulness. Another benchmark by Pirgazi et al.^7^ provided acceptable accuracy levels but here the noise levels were not varied enough to make noise-related conclusions. Other smaller benchmarks are also found in the publications of new methods^8–10^ but there both the selection of benchmarked methods and data properties are very limited. When combined together, all these benchmarks include a large amount of inference methods, yet fail to provide the community with clear guidance of the strengths and weaknesses of surveyed methods on data with different properties, which is useful for identifying the most suitable method for a particular dataset.

**Figure 1:**
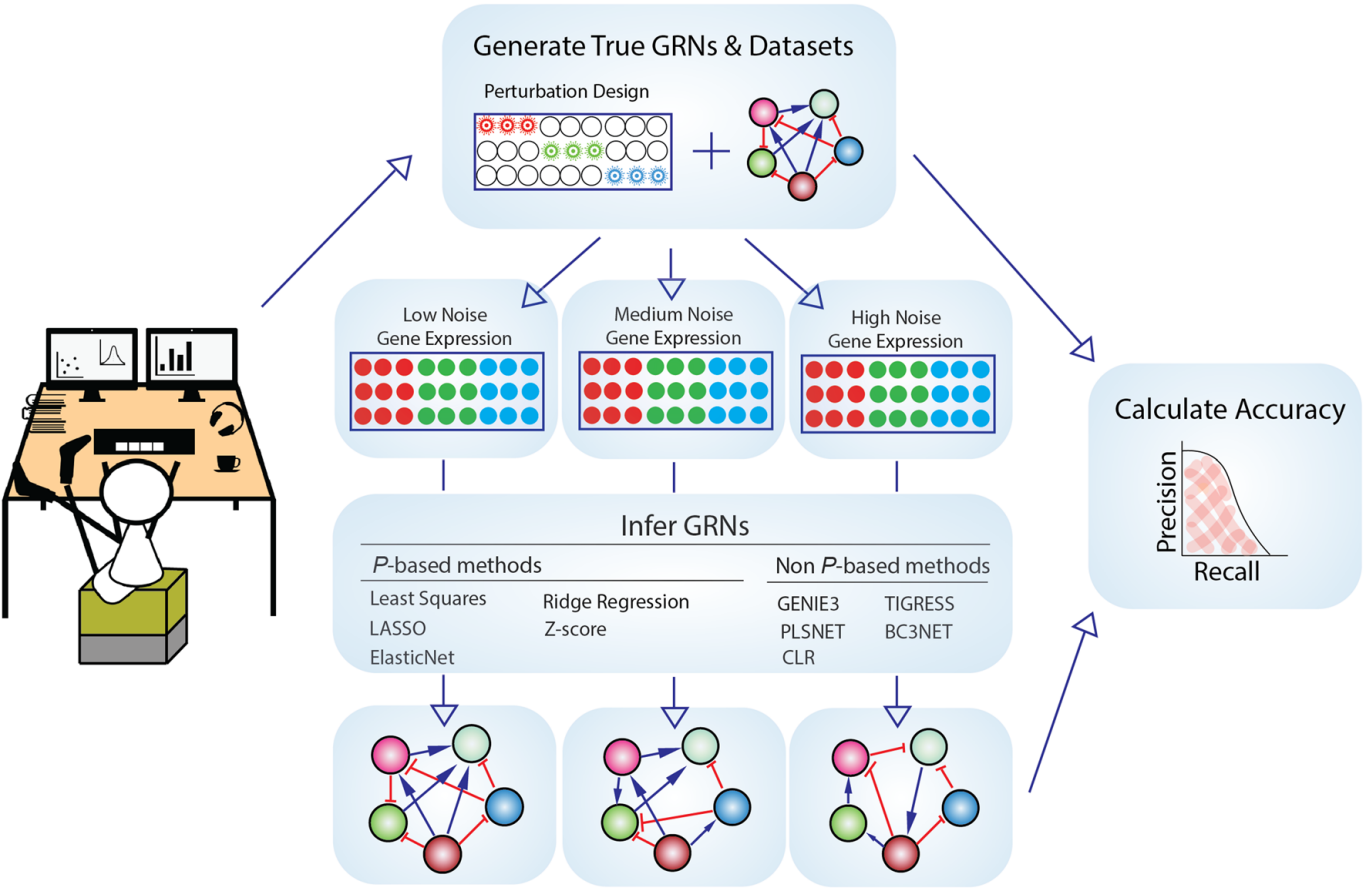
Workflow of the benchmarking pipeline. First, in silico true GRNs and perturbation-based gene expression data are generated, followed by addition of low, medium and high levels of Gaussian noise. GRNs are then inferred using ***P***-based (that use the perturbation design) and non ***P***-based (that do not use the perturbation design) inference methods, and finally the accuracy of each prediction is calculated by several measures, including area under precision-recall (AUPR).

In addition to the mentioned shortcomings of the current benchmarks, one key aspect that has not previously been examined is the importance of knowing and using the experimental perturbation design, which only some GRN inference methods are capable of. To investigate this, we benchmarked several GRN inference methods based on diverse mathematical models, divided into two categories based on whether they use knowledge of the perturbation design (***P***-based methods, where ***P*** refers to the perturbation design matrix) or not (non ***P***-based methods). Perturbation in GRN inference, and gene expression studies in general, can take many forms such as overexpression using plasmids in yeast^11^ or knockdown experiments using RNAi^12^. Regardless of how the perturbation is performed, methods that use the knowledge of the perturbation design can use this information in different ways, either as part of the system model, as prior information, or to filter data, in order to build a GRN. ***P***-based methods, by mapping the perturbations to measured gene expression, can identify the causality behind the gene regulation^13–16^, a crucial aspect in GRN inference when the ultimate goal is to identify genetic mechanisms and propose possible therapies. In contrast, most methods that do not utilize the perturbation design are limited to finding associations between genes. We applied methods that either do or do not use the perturbation design to in silico datasets generated using GeneNetWeaver^17^ and GeneSPIDER^2^ with varying noise levels. Inferred GRNs were compared to their gold standards, and their accuracy was evaluated in terms of several metrics (Fig. 1).

The results show that ***P***-based methods are significantly more accurate than non ***P***-based ones, and that only ***P***-based methods were able to reach near perfect inference accuracy.

## Results

We applied five ***P***-based and five non ***P***-based GRN inference methods to 100- and 250-gene synthetic data with three levels of Gaussian noise: high, medium, and low (Eq. (1) in “Methods”). The high noise level corresponds roughly to the noise level of biological datasets, the medium level can be achieved following a successful preprocessing approach^18^, and at the low noise level the minimum signal is equivalent to the noise, meaning that it is relatively easy to reconstruct the underlying system. For each noise level, we measured the inference accuracy across all error levels as the area under the precision-recall (AUPR) curve. To support the validity of the drawn conclusions we also calculated the area under the receiver operating characteristic (AUROC) curve, F1-score, and Matthew’s correlation coefficient (MCC) for the 100-gene datasets.

### Utilizing the perturbation design leads to more accurate GRN inference

**Figure 2:**
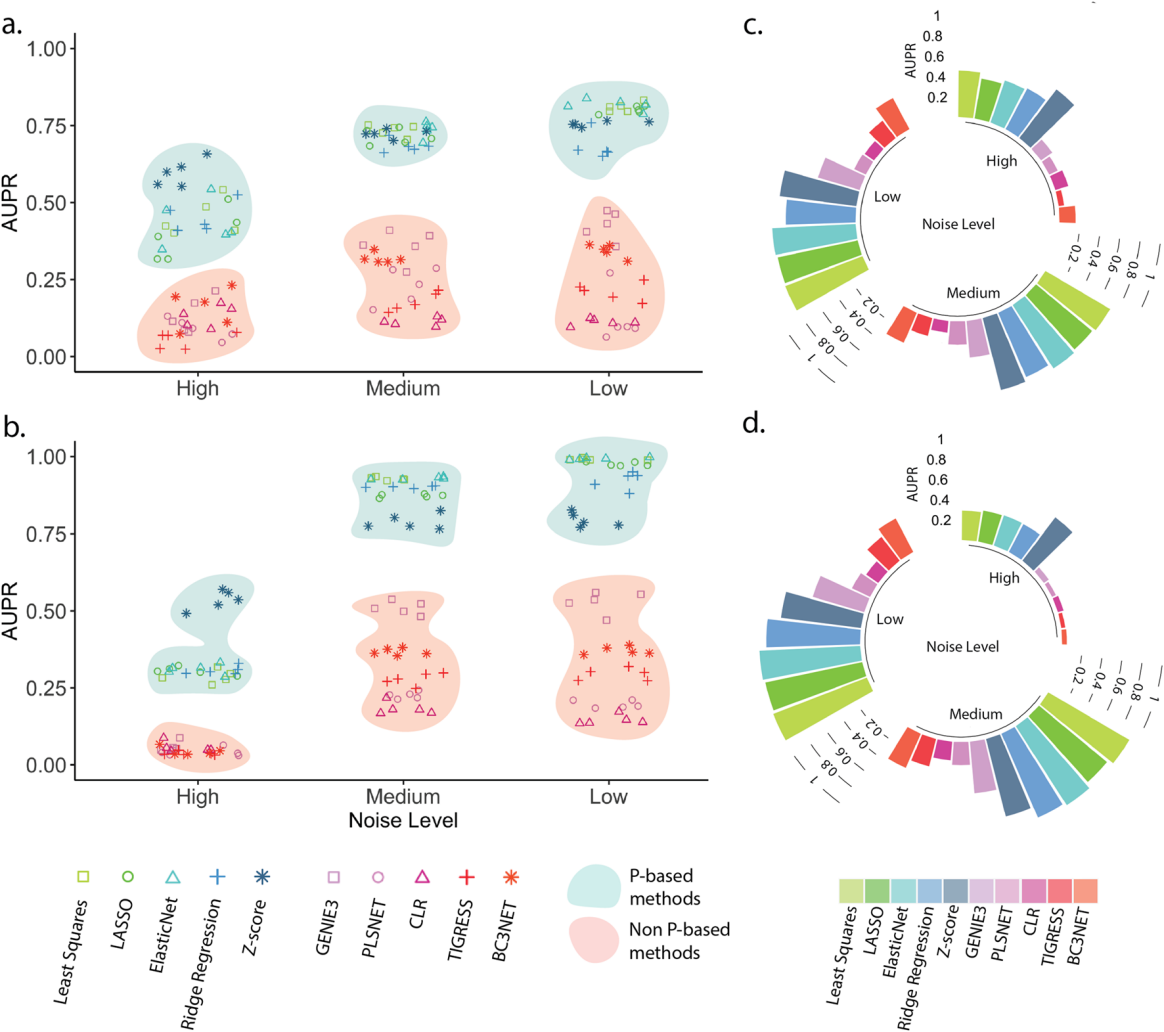
Accuracy of the GRN inference from the 100-gene synthetic datasets in terms of the area under the precision-recall (AUPR) curve. Inference accuracy in terms of AUPR from (**a**) GeneNetWeaver and (**b**) GeneSPIDER datasets. The x-axis represents different noise levels, corresponding to signal-to-noise ratio (SNR) levels 0.01, 0.1, and 1, and the y-axis denotes the AUPR levels calculated over different sparsities. Each method has five data points for each noise level for data generated from different true GRNs. The ***P***-based and non ***P***-based methods are represented by different markers and colors, and are highlighted together with blue and red, respectively. The average AUPR values of the 5 datasets are shown in circular bar plots for the (**c**) GeneNetWeaver and (**d**) GeneSPIDER datasets.

A general noise-related trend was observed in GRN inference accuracy, that AUPR levels increase relative to decreased noise especially from ‘high’ to ‘medium’ noise levels (Fig. 2; Suppl. Table S2; Suppl. Fig. S12). The increase in AUPR levels was larger for the GeneSPIDER datasets than for GeneNetWeaver, and was significant in both cases (p < 0.05). The transition from noise level ‘medium’ to ‘low’ was still significant for the ***P***-based methods, but not for the non ***P***-based methods (p > 0.05). At all noise levels a significant difference in accuracy is observed between the ***P***-based and non ***P***-based methods, where the former outperformed the latter without exception (Suppl. Table S1).

At the high noise level, Z-score^19^ was the most accurate of all methods, followed by other ***P***-based methods, both on data generated by GeneNetWeaver and GeneSPIDER. All non ***P***-based methods performed poorly at this noise level, and there was no clear winner among them. The increase in accuracy when going from ‘high’ to ‘medium’ noise is more noticeable for the GeneSPIDER data than for the GeneNetWeaver data. A smaller increase in AUPR levels was observed when the noise level decreased from ‘medium’ to ‘low’, but this is still statistically significant for the ***P***-based methods, and resulted in some of the ***P***-based methods achieving the perfect level of AUPR on the GeneSPIDER data. For ‘medium’ and ‘low’ noise levels, GENIE3^8^ was the top performer among the non ***P***-based methods, closely followed by BC3NET^20^, yet they were with no exception outperformed by the least accurate of the ***P***-based methods. PLSNET^10^ and CLR^21^ were the least accurate of all methods across all datasets.

We also calculated the maximum F1-scores (Suppl. Fig. S5) and MCC levels (Suppl. Fig. S6) on these 100-gene datasets to support the validity of our hypothesis. The same trend as for AUPR was observed with both these alternate measures, with ***P***-based methods always outperforming the non ***P***-based ones.

### Correct knowledge of the perturbation design is crucial for accurate GRN inference

**Figure 3:**
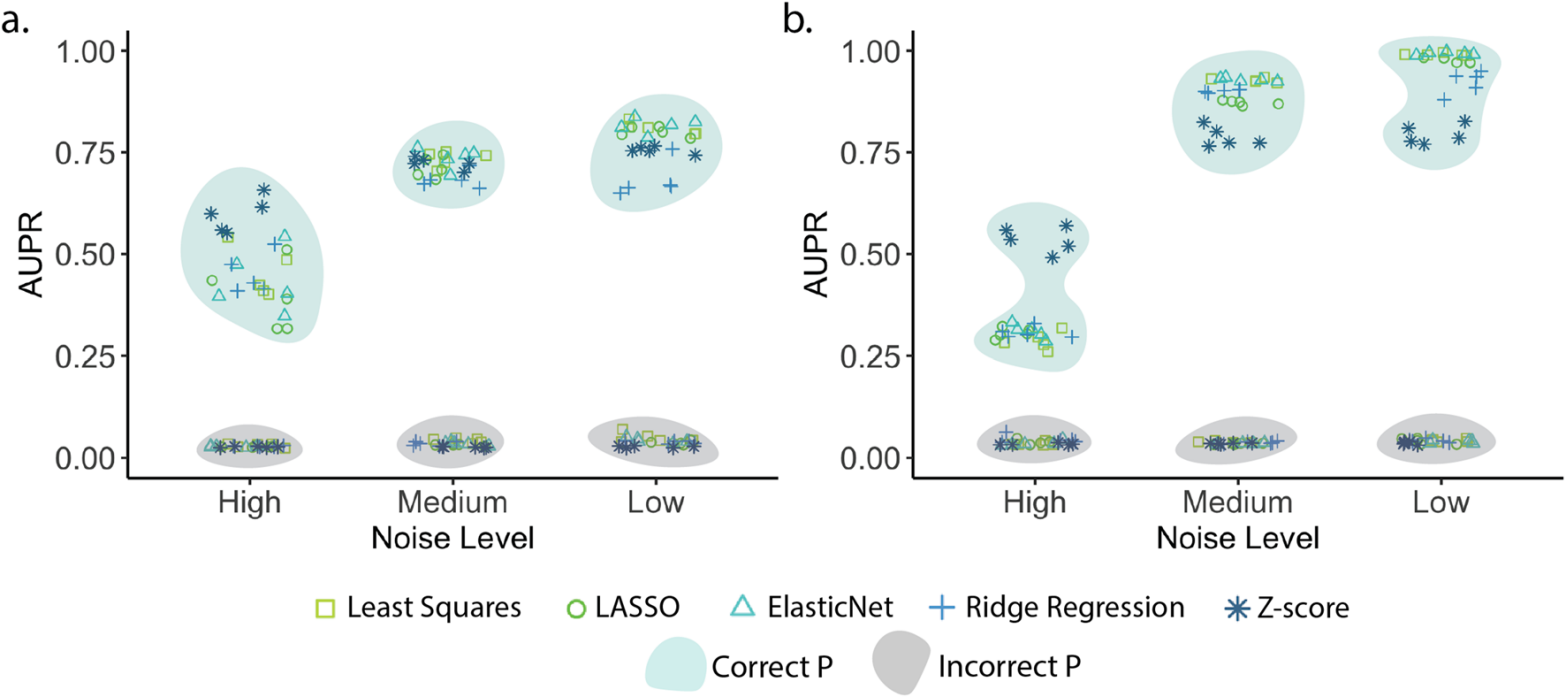
Accuracy of the ***P***-based GRN inference methods using the correct and incorrect perturbation designs with the 100-gene synthetic datasets generated by (**a**) GeneNetWeaver and (**b**) GeneSPIDER in terms of the area under the precision-recall (AUPR) curve.

To further investigate the effect of the information stored in the design matrix on inference accuracy of the ***P***-based methods, we randomly displaced every perturbation in the perturbation design matrix, and applied the ***P***-based methods to these 100-gene datasets where the connection between gene expression and its perturbation design is broken. The results showed that, regardless of any decrease in noise levels, the performance from the incorrect perturbation design remained around the random line in terms of AUPR (Fig. 3, Suppl. Table S1). This situation occurs as the ***P***-based methods are built on the assumption that the input ***P*** matrix represents the actual perturbations, and they can reach almost perfect accuracy thanks to utilizing the correct perturbation design.

### Stratification of ***P***-based and non ***P***-based methods

**Figure 4:**
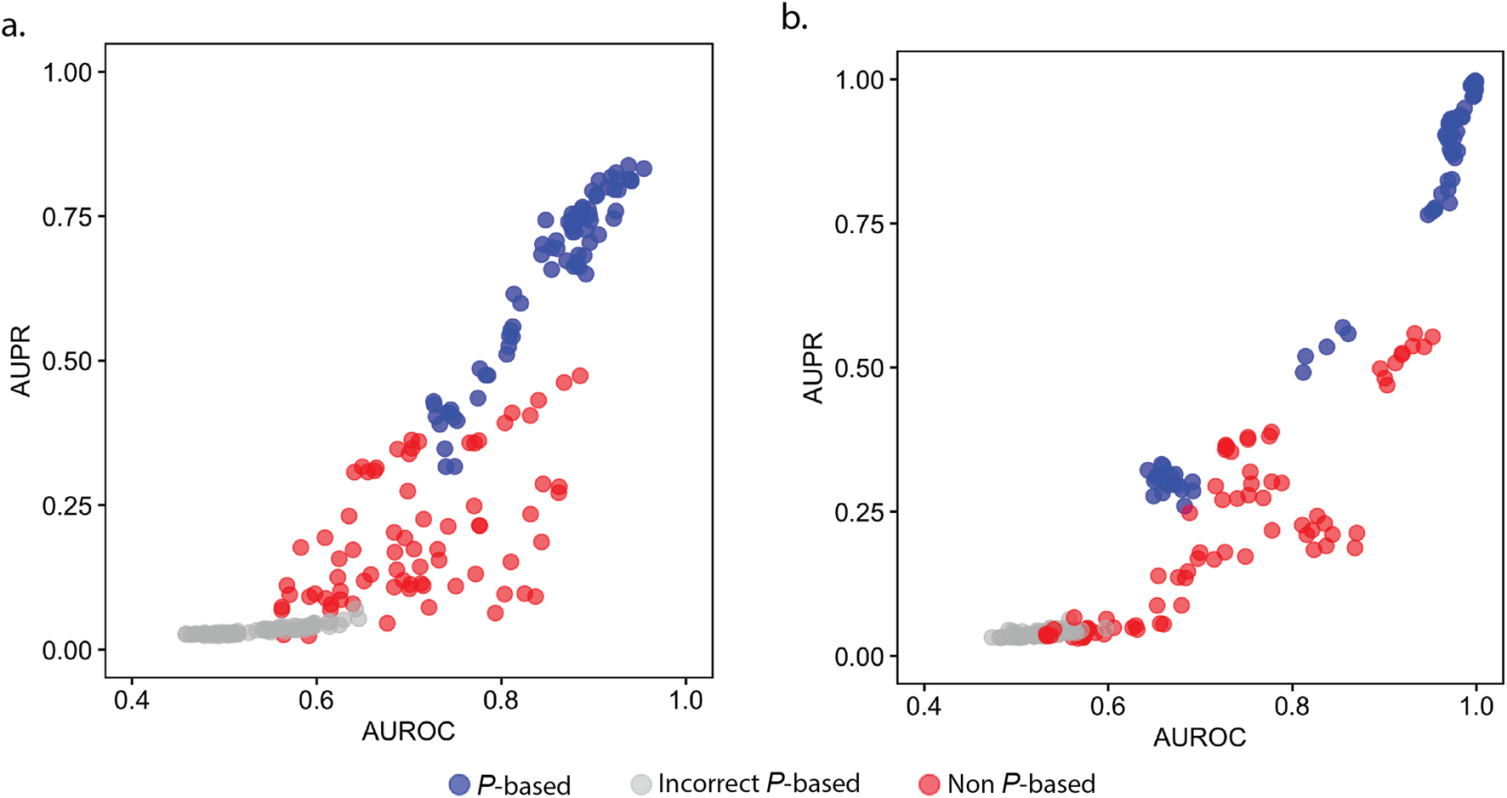
Combined accuracy of all methods on the 100-gene datasets of all noise levels based on their perturbation background. The x-axis represents the area under the receiver-operating-characteristic (AUROC) curve, and the y-axis represents the area under the precision-recall (AUPR) curve for the (**a**) GeneNetWeaver and (**b**) GeneSPIDER datasets.

We have shown examples of how ***P***-based methods outperform non ***P***-based methods under changing noise levels, and that the correct perturbation design is crucial for the ***P***-based methods to be able to perform accurately. To provide an overview of the benchmark results, we plotted the AUPR scores versus the AUROC scores for the 100-gene datasets at all noise levels (Fig. 4). This highlights the separation between ***P***-based and non ***P***-based methods, and shows that only ***P***-based methods, when provided with the correct knowledge of the perturbation design, can achieve near perfect levels of GRN inference accuracy in terms of both AUPR and AUROC, whereas the accuracy of non ***P***-based methods remains limited to a level in terms of AUPR (< 0.6) even at low noise levels (Fig. 4).

### Similarity between methods

**Figure 5:**
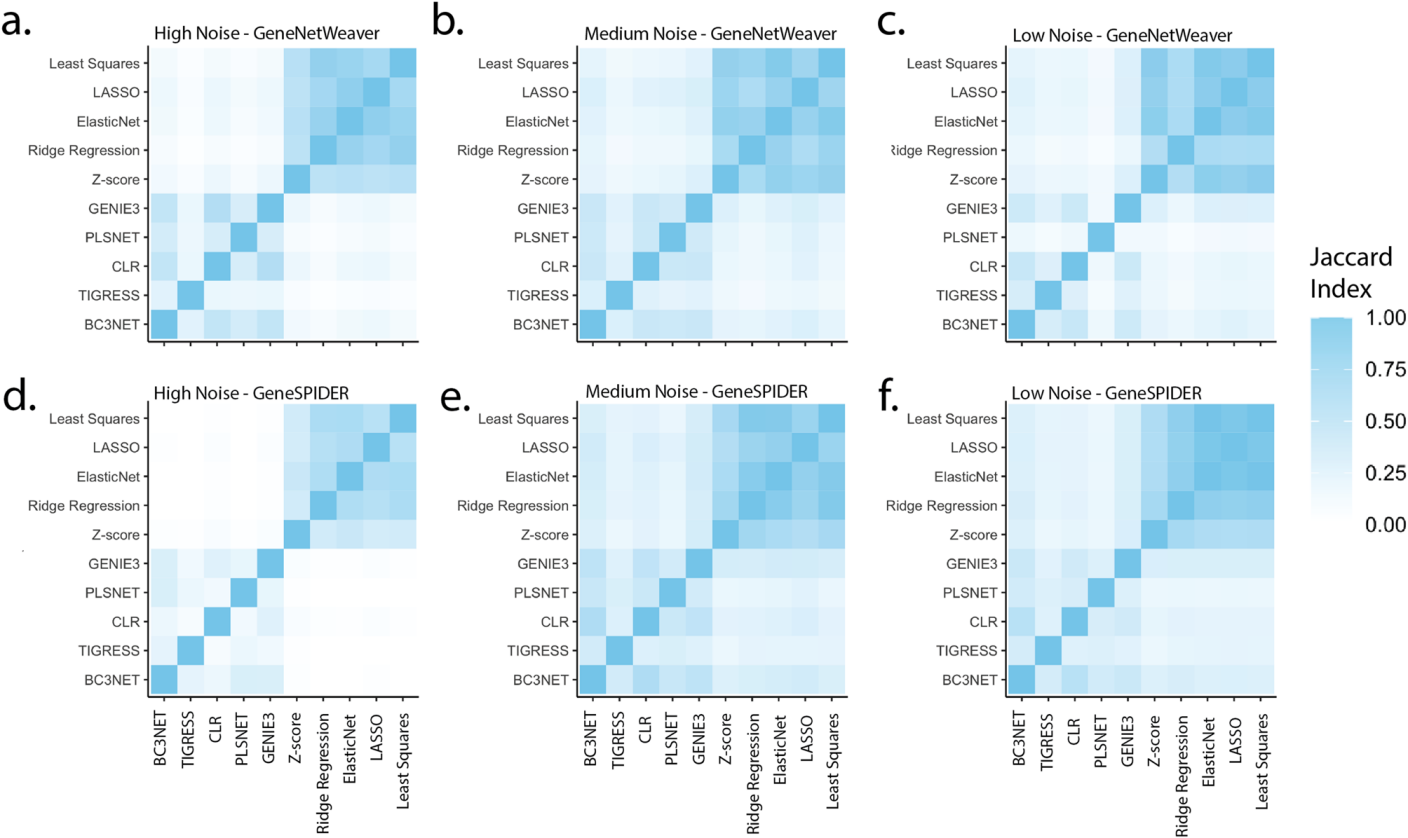
Average Jaccard index between the interactions predicted by the benchmarked methods across 5 100-gene datasets for each of the three noise levels, high (left column), medium (middle column), and low (right column) noise levels for (**a**–**c**) for GeneNetWeaver and (**d**–**f**) for GeneSPIDER data.

In order to investigate the similarity between the methods in terms of predicted interactions, we used the benchmarked results on the 100-gene synthetic data and measured the Jaccard index of the predicted edges in the maximum F1-score GRNs for each method pair, averaged for all datasets with the same properties (Fig. 5). The results show that the GRNs from ***P***-based and non ***P***-based methods cluster within the categories, where the ***P***-based methods have an average Jaccard index at all noise levels of 0.75 for GeneNetWeaver data and 0.72 for GeneSPIDER data, while the non ***P***-based methods are less similar at on average 0.33 for GeneNetWeaver data and 0.30 for GeneSPIDER data. The Jaccard indices between categories were statistically significantly different at all noise levels (Suppl. Table S3). The similarity between ***P***-based and non ***P***-based methods is very low at high noise (0.09 and 0.02 on average for the GeneNetWeaver and GeneSPIDER data, respectively) but increases for the lower noise levels.

We also calculated the fraction of true interactions for each method pair, *i.e.* the portion of the overlap between two methods found in the true GRN (Suppl. Fig. S9). One minus this fraction would accordingly give the agreement on false positives. The overall trend followed the trend in method prediction overlap (Fig. 5), where the fraction of true edges is higher for the overlap between the ***P***-based methods than between the non ***P***-based. The fraction of true edges remained considerably lower for the 100-gene GeneNetWeaver datasets across all noise levels than for the 100-gene GeneSPIDER datasets. For the GeneNetWeaver datasets at high noise level, the highest true fraction was observed for the overlap between the non ***P***-based methods, but this was not the case for the other noise levels. For the GeneSPIDER datasets, the true fraction was always considerably higher for the overlap between the ***P***-based methods, and showed an increasing trend with decreasing noise.

### Speed benchmark

**Figure 6:**
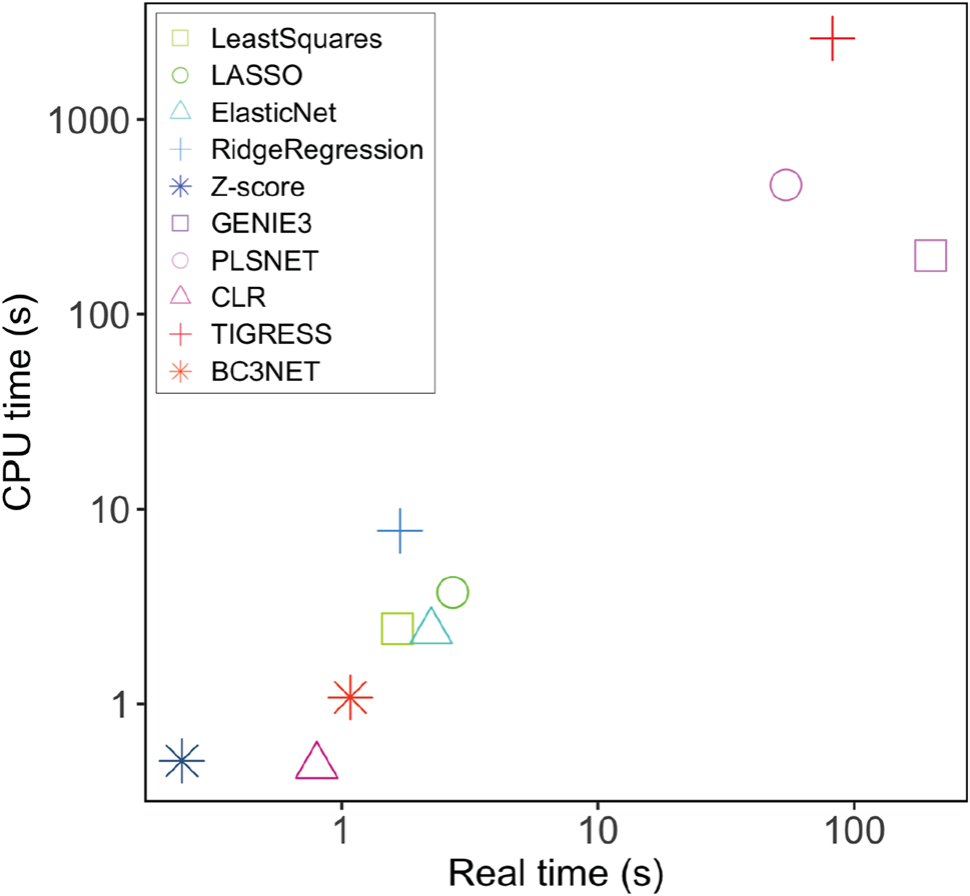
Speed benchmarking of the GRN inference methods on a 100-gene dataset. The x-axis shows the real time and the y-axis the CPU time per GRN inference run in seconds.

Among all benchmarked methods, Z-score and CLR were the fastest in CPU time, but Z-score was faster in real time, followed by BC3NET in both CPU and real time. TIGRESS^21^ was by far the slowest, followed by PLSNET and GENIE3, in CPU time. In real time, however, GENIE3 was the slowest because TIGRESS and PLSNET use MATLAB’s parallelization (Fig. 6, Suppl. Table S5).

### The effect of selfloops on GRN inference accuracy

A fundamental difference that we observed between the benchmarked ***P***-based and non ***P***-based methods is that the former infers selfloops while the latter does not. Selfloops represent the rate of gene transcript degradation, which is an important parameter for the system’s stability, making them an essential part of the true network. To make a fair assessment of both method categories, we included the selfloops in the true networks for the ***P***-based methods, and removed them from the true network for the non ***P***-based methods. As GeneNetWeaver networks do not contain all selfloops, the missing ones were added for ***P***-based methods. This procedure prevents ***P***-based methods to suffer from false positives, and non ***P***-based methods to sacrifice accuracy due to false negatives. Since the main assessment of GRN inference accuracy is made mainly in terms of AUPR, both errors have an equal effect on the inference accuracy. In order to investigate the specific effect of selfloops on GRN accuracy, we removed them both from the true network and the inferred GRNs of the 100-gene datasets for all methods, including the ***P***-based ones, and observed that even though selfloops are responsible for a large part of the accuracy under high noise levels, they don’t have any effect on accuracy for medium and low noise levels (Suppl. Fig. S10). One exception is the Z-score method whose accuracy is not strongly affected by the treatment of selfloops at any noise level.

### Benchmarking on biological data from DREAM5

The fifth round of DREAM^5^ includes a GRN inference challenge based on perturbation-induced expression data for *E. coli*, and RegulonDB as the gold standard GRN. We identified a subset of this dataset (network3)^5^ with known-target knockout or overexpression perturbations, i.e. with a known ***P*** matrix, and performed GRN inference by all methods that were run on the synthetic data above. Note that most methods that participated in DREAM5 performed very poorly in this challenge as no method scored an AUPR above 0.15. However, on the known-target subset, the ***P***-based methods were able to achieve considerably higher AUPR levels, ranging between 0.30 and 0.38 (Suppl. Fig. S11). This was not the case for the non ***P***-based methods, which only reached AUPR levels between 0.01 and 0.03, which is even lower than in the DREAM5 challenge. For instance, GENIE3 achieved an AUPR of ~ 0.10 on the full challenge dataset^5^ but on the selected subset only 0.03. Taken together, this agrees with the previous results suggesting that knowing and using the ***P*** matrix can augment GRN inference accuracy considerably.

## Discussion

Previous benchmarking of GRN inference methods have provided a broad perspective of the accuracy that is achievable with different mathematical approaches on different datasets. However, a very important aspect has so far been missed, namely the type of information that the methods utilize. This study for the first time assesses the importance of using the perturbation design for accurate GRN inference. All conclusions in this study are based on the presented results which were obtained by using data that inherently represent the targeted perturbation steady-state condition (knock-down), and inference methods developed for this type of data.

As expected, a lower noise level in the data generally led to higher accuracy. For all noise levels, there was however a clear separation between ***P***-based and non ***P***-based methods in that the former consistently outperformed the latter. Furthermore, ***P***-based methods were able to achieve almost perfect accuracy, whereas the accuracy of the non ***P***-based methods remained below an AUPR of 0.6. ***P***-based methods have previously been shown to continuously improve with decreasing noise and achieve perfect accuracy under good data conditions^1,2^, supporting the present results. This suggests that, if the informativeness of real data can be improved by reducing the noise level of the system from high to at least medium via for instance preprocessing approaches^18,22^, close to perfect accuracy can be achieved by ***P***-based methods that use the knowledge of the perturbation design. As a result, accurate and reliable prediction of gene regulatory interactions could be performed to identify novel regulatory mechanisms and treatment targets, which would not be possible with the non ***P***-based methods whose accuracy is considerably lower due to not utilizing the essential knowledge of the perturbation design.

We have tested the effect of the correct perturbation information on the performances of the ***P***-based methods by misplacing every perturbation in the design matrix and breaking the connection between the gene expression and its design, which resulted in the same accuracy as random predictions. This shows that the ***P***-based methods are very powerful with the correct and complete design information, but also potentially vulnerable to errors in the design matrix, which could have either experimental or data processing causes. The value of using the design matrix was previously shown in the DREAM5 network inference challenge^5^, where inference methods using it were better able to predict the targets of transcription factors. Despite utilizing the perturbation design, these methods were not able to achieve high accuracy levels on the DREAM5 data because there only transcription factors were perturbed, which is a small fraction of all genes. This is different from the data in this benchmark where a majority of the genes in the system are perturbed, which makes highly accurate GRN reconstruction possible unless the noise level is too high. The effect of an imperfect ***P*** matrix has previously been explored^22^, where it was shown that the connection between the intended perturbation design and measured gene expression may be broken due to high noise levels or off-target effects of perturbations, leading to an incorrect mapping of the ***P*** matrix to gene expression and lower accuracy. ***P***-based methods using a ***P*** matrix that was inferred from the measured gene expression were shown to perform better than when the intended ***P*** was used, providing additional support for the importance of utilizing the correct ***P*** that suits the data.

A drawback of ***P***-based methods is that they are not possible to apply to data without a targeted perturbation design matrix, which limits their application. Non ***P***-based methods, on the contrary, are possible to apply to both targeted and untargeted perturbation data, which increases their generality but comes with a great sacrifice of accuracy. This situation can simply be seen as a tradeoff between generality and higher accuracy, where both method categories sacrifice one or another.

Given the low GRN accuracies of inference methods for data from both GeneNetWeaver and GeneSPIDER, especially at high noise levels, it is clear that only a portion of the true regulatory interactions was captured by each method. The Jaccard index of the overlap between the interactions inferred by method pairs shows that the agreement between the non ***P***-based methods is considerably lower compared to the ***P***-based methods. Combining these two results, one possible reason for such discrepancy could be that the differences in the mathematical backgrounds of the non ***P***-based methods may capture different patterns in the data, resulting in low overlap in the top inferred interactions. In contrast, ***P***-based methods not only share a larger overlap but also the fraction of true interactions is higher compared to the non ***P***-based methods, at least for GeneSPIDER data. This is most likely due to the fact that the penalized regression methods such as LASSO, ElasticNet and Ridge regression are all related to least squares and therefore are more likely to capture the same patterns in the data. It is noteworthy that the predictions of ***P***-based methods can be combined due to the large fraction of true interactions in their overlaps, and in the absence of a true GRN, the intersection of these methods is likely to provide the best GRN that can be obtained.

Autoregulation is of crucial importance for a system’s stability. Therefore, all synthetic true networks we generated contain selfloops to effectuate autoregulation, and the data generated from these networks are partly based on selfloops. The two categories of inference methods however follow different approaches regarding the selfloops as the ***P***-based methods tend to always infer these, while the non ***P***-based methods do not. Not including the selfloops in the true GRN would result in false positives for the ***P***-based methods, and including them would result in false negatives for the non ***P***-based methods. Our solution to this situation is to treat both categories in a fair way by keeping all selfloops in the true GRN for the ***P***-based methods, and removing them from the true GRN when comparing to the inferred GRNs of the non ***P***-based methods. Since the accuracy assessment was mainly done by AUPR, this selfloop treatment has the same effect on both situations as false positives and false negatives have equal roles in AUPR. We also explored an alternative approach to remove the selfloops from both the true GRN and the inferred GRNs of the ***P***-based methods. This had little effect at the low and medium noise levels, but at high noise most of the accuracy was lost compared to the standard benchmarking method, for all methods except Z-score that only lost about half the AUPR.

Without considering selfloops, Z-score stands out as much more accurate than all other methods at high noise levels. Also when considering selfloops, the most reliable method for high noise was Z-score. Previous studies have shown similar results where the Z-score approach is among the top performing methods despite its simplicity^17,19,23,24^. Although it is the winner at the most difficult noise level, Z-score is outperformed by the other ***P***-based methods at medium and low noise on the GeneSPIDER data. It can thus be either the best or the worst of the ***P***-based methods depending on the conditions.

We observed a strange situation with the direction of interactions inferred by Genie3 in the benchmark, where the reverse edge direction was much more accurate than the original direction (Suppl. Note 1). This was however not the case for the results from the DREAM4 in silico multifactorial network inference challenge. Our analysis suggests that the reverse direction generally gets the highest weight between regulators, which results in a much higher accuracy when the true GRN has a large fraction of regulators, as in this benchmark (Suppl. Fig. S14). According to the Genie3 authors, it yields the wrong link direction when applied to single gene knockdown data, which is used here (P. Geurts, personal communication). Note that even when reversing the direction of the Genie3 GRNs to optimize its performance, it could not compete with the worst performing ***P***-based method in any dataset generated for this benchmark. As the effect of the link direction depends on the data, we encourage other researchers to investigate if they can verify our findings with their own data.

A large portion of GRN inference methods infer directed interactions where an edge is drawn from a regulator gene to its target(s). Some of these methods also assign a sign to these interactions to indicate activation or inhibition. Some methods, however, only infer undirected interactions, where the source and the target genes are unknown, and the networks are symmetric. The accuracy calculation in this study was performed by considering the direction of the interactions but not their sign. The direction of an interaction is of crucial importance in a GRN, but two of the benchmarked methods in this study, CLR and BC3NET, infer the interactions without direction, i.e. all inferred links are in both directions. While this will degrade the performance of undirected methods in a directed benchmark, it is not obvious how to avoid it. In a real situation one might derive a direction from regulators to targets, but providing this extra information in a benchmark would give these methods an unfair advantage. Another aspect of the present benchmarking method is that, ignoring the sign of the interactions could potentially result in a GRN to be rated perfectly accurate although all the links have the wrong sign. However, given that the sign of the interaction is not inferred by any of the non ***P***-based methods, we did not include the sign of the predicted links in the benchmark, even though this gives such methods an advantage.

In this study we performed a broad benchmark with a novel idea of comparing the mathematical model category of the GRN inference methods, and showed that the knowledge of the perturbation design is essential for accurate GRN inference, and that the methods utilizing this information are significantly more accurate and reliable than the ones not utilizing it. Given that one of the biggest aims of GRN inference is to reveal unknown mechanisms that may become helpful in better understanding and treating genetic diseases, this study which demonstrates the positive effect of using the perturbation design on GRN accuracy may lead to a significant change in the field both in terms of biological data generated and methods developed. Therefore, based on our presented results, we strongly recommend experimentalists to perform targeted perturbation experiments, and computational systems biologists to use and develop perturbation-based methods for more accurate and reliable GRN inference, especially when the ultimate goal is to infer novel regulatory interactions as treatment targets where false predictions would lead to wasted efforts.

## Materials and methods

### Networks and datasets

We have generated synthetic networks and datasets via GeneNetWeaver and GeneSPIDER for benchmarking the gene regulatory network inference methods. We also extracted a known-target perturbation subset from the *Escherichia coli* dataset (network 3) of the DREAM5^5^ network inference challenge.

#### In silico true network generation via GeneNetWeaver

Five subnetworks of 100-genes were extracted from the complete *E. coli* network. All genes were requested to be regulators but GeneNetWeaver does not assign the exact requested number, resulting in a varying number of regulators per subnetwork. The vertices were drawn randomly with the “greedy” edge selection. The true sparsity of the 5 GRNs, without selfloops, ranges between 1.48 and 1.95 links per gene.

#### In silico true network generation via GeneSPIDER

Five synthetic networks were generated in scale-free topology with directed and signed edges. Each gene is allowed to be a regulator, and on average three links per node were assigned. The true sparsity of the 5 GRNs, without selfloops, ranges between 2.22 and 2.38 links per gene.

#### In silico perturbation design

To be able to observe the regulatory effect of a gene on one or more others, it is important to introduce alterations to the system. These alterations are called “perturbations”, which can be applied to all genes in the system or target specific genes, one at a time. Some GRN inference methods can infer GRNs from measurements of both types of perturbations (called non ***P***-based in this study), while some methods require known targeted perturbations (***P***-based). To investigate the importance of the knowledge of targeted perturbations in GRN inference, we generated single target-perturbation matrices with three replicates per perturbation experiment, to be later used as the input cue to the true regulatory system when generating perturbation-based data from it. The perturbation information is stored in a binary N-by-M matrix, where N refers to genes and M experiments, assigning − 1 (for knockdown) to all perturbations and 0 to all other cells. This matrix is throughout the paper referred to as the ***P*** matrix.

#### In silico noise-free data generation via GeneNetWeaver

For each of the five subnetworks, a noise-free gene expression dataset of steady-state knockdown perturbations was generated from ordinary differential equations. No normalization was performed, and noise-free fold changes were calculated by the log_2_ ratio between the gene expression and its wild type value. The fold change matrix was transposed and replicated three times, to simulate a perturbation experiment with three replicates. The resulting noise-free fold change gene expression matrix is therefore in size 100 × 300 (genes × experiments).

#### In silico noise-free data generation via GeneSPIDER

For each of the five synthetic networks a noise-free fold change gene expression dataset with three replicates was generated. Unlike GeneNetWeaver, GeneSPIDER directly generates fold changes instead of generating gene expression and wild type separately. GeneSPIDER also inherently allows for replicates, therefore no manual replication was necessary. The resulting noise-free fold change gene expression matrix is in size 100 × 300 (genes × experiments).

### Noise generation

Noise was generated in the same way for the data from both generation tools to allow for a fair comparison based on the signal-to-noise ratio (SNR). Given a target SNR, we used Eq. (1) to calculate the required variance (λ). Then we generated a random noise matrix of the same data size with the desired SNR using the derived variance.

1 $$SNR = \frac{min\left(svd\left(X\right)\right) }{\sqrt{{\chi }^{-2}\left(1-\alpha , NM\right)\lambda }}$$

In Eq. (1), *svd(X)* is a set of values from the singular value decomposition of the noise-free fold change gene expression matrix *X*, 1 − α is the confidence level, 0.99, N is the number of genes, and M is the number of experiments. λ refers to the variance. Following this approach, we generated three different noise matrices of ‘high’, ‘medium’, and ‘low’ noise levels from SNR levels of 0.01, 0.1, and 1, respectively. The generated noise matrices were added to their corresponding noise-free gene expression matrix to have the noisy data to perform GRN inference.

### DREAM5 *E. coli* subset

A known-target perturbation subset was extracted from the DREAM5 *E. coli* (network3) challenge dataset^5^ using the mapping file called “chip features” where the “DeletedGenes” and “OverexpressedGenes” columns refer to knockout and overexpression experiments, respectively. The entries with the same experiment number without any perturbation information were considered the control experiments, and their average was used to calculate the fold change gene expression. Knockdown experiments were discarded since their targets were not specified. This subset is in size 41 × 193 (genes × experiments) while the full dataset is 4297 × 805.

### Benchmarked GRN inference methods

We investigated several methods from different mathematical backgrounds, i.e., regression, mutual information, random forests and Bayesian, and gathered the state of the art methods from each mathematical background. The mutual information based methods CLR and BC3NET were selected because they performed well in the DREAM network inference challenges. Note that in the DREAM competition, the ***P***-based methods used here were not used with a ***P*** matrix as input. ***P***-based methods such as LASSO and related regression-based methods were chosen because they are well known and commonly used in the field. To complement these we chose the Z-score approach for ***P***-based observed effect GRN inference.

#### Least squares

The least square regression provides the optimal fit between the dependent variable and the independent variables by minimizing the sum of squared residuals. Assuming at steady state ***YA*** + ***P*** = 0 ^1^, the GRN ***A*** is estimated as − ***P*** × ***Y***^†^, where ***Y*** is the observed expression matrix, ***P*** the perturbation design matrix, and ^†^ denotes the Moore–Penrose inverse. ***P*** contains a − 1 in the experiment/gene cell if it is a knock-down perturbation, and + 1 for overexpression (not used here). Noise terms are modeled implicitly here.

#### ElasticNet^25–27^

ElasticNet is a regression model that combines LASSO’s L1 regularization parameter with the L2 penalty from the Ridge regression to overcome LASSO’s limitations especially when the data is ill-conditioned. We used Matlab’s *Glmnet* implementation with α = 0.7, which corresponds to ElasticNet (0 < α < 1).

#### LASSO^26,27^

Least absolute shrinkage and selection operator (LASSO) is a regression-based variable selection and regularization method, which utilizes the perturbation design and is used here for accurate prediction of gene regulatory interactions. LASSO uses the L1 regularization parameter. We used Matlab’s *Glmnet* implementation with α = 1, which corresponds to LASSO.

#### Ridge regression^25–27^

Ridge regression is a regression method that uses the L2 penalty to estimate the regression coefficients of highly correlated explanatory variables. We used Matlab’s *Glmnet* implementation with α = 0.

#### Z-score^19^

Z-score corresponds to the distance between an observed gene expression and the mean of the gene sample that it is compared to, divided by the standard deviation of the same sample. In this study, we implemented a Z-score-based approach that utilizes the perturbation design matrix. Each Z-score value is considered as a weight between the gene and the intended target of the perturbation.

#### GENIE3^8^

GENIE3 uses random forests-like tree ensemble methods to build weighted and directed unsigned interactions of a gene against the others. It uses gene expression profiles without requiring knowledge of the perturbation design. The weights in the output GRN correspond to the strength of the regulation from the regulator gene to its target. GENIE3 outperformed its competitors in DREAM4 in silico network inference challenge, however it is computationally expensive due to its tree-based algorithm. We used GENIE3 in Matlab with its default parameters, which are random forests with 1000 trees at each step. We tested using the output in two ways for the default parameters when all genes are assumed as transcriptional regulators: either assuming that regulators are in rows or in columns. As the latter gave much higher accuracy, this is how we used the output (Suppl. Fig. S14).

#### PLSNET^10^

PLSNET uses the partial least squares approach to construct weighted, directed but unsigned gene regulatory networks. It does not utilize the perturbation design. We used PLSNET in Matlab with its default parameters, which are *‘nfac’* = 5, *‘K’* = 20, *‘T’* = 1000.

#### CLR^21^

Context likelihood of relatedness (CLR) uses mutual information in its background, and outputs weighted but undirected and unsigned gene regulatory networks. We used the settings *‘method’* = ‘rayleigh’, *‘n’* = 10, *‘k’* = 3.

#### TIGRESS^9^

TIGRESS is a regression-based gene regulatory network inference method that outputs directed but unsigned networks where the weights correspond to the strength of the regulation. We used default settings except *'R'* = 1000 as the default of 10,000 was too slow.

#### BC3NET^20^

BC3NET is a Bayesian bootstrapped mutual information-based gene regulatory network inference method that outputs unsigned and undirected but weighted networks, where the edge weights denote the ensemble consensus rate in terms of the corresponding mutual information. We used its R implementation and default parameters (100 bootstraps).

### Accuracy calculation and metrics

Accuracy of GRN inference is evaluated mainly in terms of the area under the precision-recall (AUPR) curve, but also area under the receiver-operating-characteristic (AUROC) curve, Matthew’s correlation coefficient (MCC), and the F1-score. Even though the true GRNs are signed, and some of the benchmarked methods also infer signed interactions, the sign of the interaction was not used in the accuracy calculation, but the direction was.

2 $$TPR=\frac{TP}{TP+FN}$$

3 $$FPR=\frac{FP}{FP+TN}$$

4 $$Precision=\frac{TP}{TP+FP}$$

5 $$MCC=\frac{TP*TN-FP*FN}{\sqrt{\left(TP+FP\right)\left(TP+FN\right)\left(TN+FP\right)\left(TN+FN\right)}}$$

6 $$F1-score=2*\frac{precision*recall}{precision+recall}$$

In Eqs. (2)–(5), TP, FP, TN, and FN refer to the total number of true positives, false positives, true negatives, and false negatives, respectively. In Eq. (2), TPR denotes the true positive rate, or recall. In Eq. (3), FPR denotes the false positive rate. In Eq. (5), MCC refers to Matthew’s correlation coefficient.

#### Area under the precision-recall (AUPR) curve

On a coordinate plane, the true positive rates (recall) (Eq. 2) are placed on the horizontal axis (x-axis) and the precision values (Eq. 4) are placed on the vertical axis (y-axis) for different sparsity levels that form a curve from the top of the y-axis where the precision equals to 1 and recall to 0 to the right of the x-axis where the recall equals to 1 and precision to 0. The area trapped under this curve is called the AUPR, and its value is between 0 and 1 where the former refers to a random performance whereas the latter denotes perfection.

#### Area under the receiver-operating characteristic (AUROC) curve

On a coordinate plane, the false positive rates (Eq. 3) are placed on the horizontal axis (x-axis) and the true positive rates (Eq. 2) are placed on the vertical axis (y-axis) for different sparsity levels that form a curve from the top right corner of the system where both FPR and TPR equal to 1, to the bottom left corner of the system where both values equal 0. The area trapped under this curve is called the AUROC, and its value is between 0 and 1 where the former refers to a fully misclassified system whereas the latter denotes perfection. A random performance is at AUROC 0.5.

#### Matthew’s correlation coefficient (MCC)

MCC (Eq. 5) is an accuracy measurement that takes all predictions (true positives, false positives, true negatives, and false negatives) into account to calculate correlation coefficients between the true and predicted values. MCC is commonly used in the field, and a trusted quantity known to not be affected by class imbalance. It ranges between − 1 and 1, where the former refers to a complete misclassification and the latter denotes a perfect classification. A random prediction has an MCC of 0.

#### F1-score

The F1-score (Eq. 6) is an accuracy measure based on precision and recall. It is preferred over MCC in some situations where there is an uncertainty regarding the true negatives, i.e. whether they are actual negatives or yet unknown. It ranges between 0 and 1, where the former occurs when either the precision or recall is 0, and the latter implies perfect prediction.

#### Sparsity selection approach

We applied two different approaches, one for the penalty-based methods (LASSO, ElasticNet, and Ridge regression), and one for all the others. The penalty-based methods infer an independent GRN for each value in the input penalty vector, meaning each inferred GRN should be treated as its own. For these methods, we inferred 30 GRNs whose sparsity ranges from full to empty using logspace(− 6, 0, 30). The accuracy in terms of AUPR is calculated across these 30 GRN accuracy points. For the other methods, since they either output a fully connected GRN of weighted interactions, or they produce a single network with an optimized sparsity, i.e. BC3NET, we used every unique value in the GRN as a cutoff and reduced the GRN for each of these unique values. For 100 gene networks, this means 100 K data points were considered in the GRN accuracy calculation (for the ones which output a fully connected GRN). Each GRN of a different sparsity is contained in the initial output of the method unlike the penalty-based methods.

### Speed benchmark

The benchmarked methods were run on a computer with 16 Intel Xeon E5620 2.40 GHz CPUs and 70 GB of RAM.

## Supplementary Information

Supplementary Information.

## Data Availability

The datasets supporting the conclusions of this article, and the code used to generate the results presented in this article are available in https://bitbucket.org/sonnhammergrni/benchmark/.

## References

[1] Tjärnberg A, Nordling TEM, Studham M, Nelander S, Sonnhammer ELL (2015). Avoiding pitfalls in L1-regularised inference of gene networks. Mol. Biosyst..

[2] Tjärnberg A, Morgan DC, Studham M, Nordling TEM, Sonnhammer ELL (2017). GeneSPIDER—Gene regulatory network inference benchmarking with controlled network and data properties. Mol. Biosyst..

[3] Madar A, Greenfield A, Vanden-Eijnden E, Bonneau R (2010). DREAM3: Network inference using dynamic context likelihood of relatedness and the inferelator. PLoS ONE.

[4] Greenfield A, Madar A, Ostrer H, Bonneau R (2010). DREAM4: Combining genetic and dynamic information to identify biological networks and dynamical models. PLoS ONE.

[5] Marbach D (2012). Wisdom of crowds for robust gene network inference. Nat. Methods.

[6] Bellot P, Olsen C, Salembier P, Oliveras-Vergés A, Meyer PE (2015). NetBenchmark: A bioconductor package for reproducible benchmarks of gene regulatory network inference. BMC Bioinform..

[7] Pirgazi J, Olyaee MH, Khanteymoori A (2021). KFGRNI: A robust method to inference gene regulatory network from time-course gene data based on ensemble Kalman filter. J. Bioinform. Comput. Biol..

[8] Huynh-Thu, V. A., Irrthum, A., Wehenkel, L. & Geurts, P. Inferring regulatory networks from expression data using tree-based methods. *PLoS One* **5**, (2010). 10.1371/journal.pone.0012776 PMC2946910 20927193

[9] Haury A-C, Mordelet F, Vera-Licona P, Vert J-P (2012). TIGRESS: Trustful Inference of gene REgulation using stability selection. BMC Syst. Biol..

[10] Guo S, Jiang Q, Chen L, Guo D (2016). Gene regulatory network inference using PLS-based methods. BMC Bioinform..

[11] Prelich G (2012). Gene overexpression: Uses, mechanisms, and interpretation. Genetics.

[12] Han H (2018). RNA interference to knock down gene expression. Methods Mol. Biol..

[13] Pearl J (2015). An Introduction to Causal Inference.

[14] Meinshausen N (2016). Methods for causal inference from gene perturbation experiments and validation. Proc. Natl. Acad. Sci. USA..

[15] Ud-Dean SMM, Gunawan R (2014). Ensemble inference and inferability of gene regulatory networks. PLoS ONE.

[16] Ud-Dean SMM, Gunawan R (2016). Optimal design of gene knockout experiments for gene regulatory network inference. Bioinformatics.

[17] Schaffter T, Marbach D, Floreano D (2011). GeneNetWeaver: In silico benchmark generation and performance profiling of network inference methods. Bioinformatics.

[18] Seçilmiş D (2020). Uncovering cancer gene regulation by accurate regulatory network inference from uninformative data. NPJ Syst. Biol. Appl..

[19] Prill RJ (2010). Towards a rigorous assessment of systems biology models: The DREAM3 challenges. PLoS ONE.

[20] de Matos Simoes R, Emmert-Streib F (2012). Bagging statistical network inference from large-scale gene expression data. PLoS ONE.

[21] Faith JJ (2007). Large-scale mapping and validation of *Escherichia coli* transcriptional regulation from a compendium of expression profiles. PLoS Biol..

[22] Seçilmiş D, Hillerton T, Nelander S, Sonnhammer ELL (2021). Inferring the experimental design for accurate gene regulatory network inference. Bioinformatics.

[23] Aalto A, Viitasaari L, Ilmonen P, Mombaerts L, Gonçalves J (2020). Gene regulatory network inference from sparsely sampled noisy data. Nat. Commun..

[24] Maetschke SR, Madhamshettiwar PB, Davis MJ, Ragan MA (2014). Supervised, semi-supervised and unsupervised inference of gene regulatory networks. Brief. Bioinform..

[25] Zou H, Hastie T (2005). Regularization and variable selection via the elastic net. J. R. Stat. Soc. Series B (Stat. Methodol.)..

[26] Friedman J, Hastie T, Tibshirani R (2010). Regularization paths for generalized linear models via coordinate descent. J. Stat. Softw..

[27] Tibshirani R (1996). Regression shrinkage and selection via the lasso. J. R. Stat. Soc. Series B Stat. Methodol..

