# Supplementary material for: Knowledge of the perturbation design is essential for accurate gene regulatory network inference
Source: Sci Rep. 2022 Oct 3;12:16531. doi: 10.1038/s41598-022-19005-x (PMC9529923; doi:10.1038/s41598-022-19005-x)
Supplement: Supplementary file 1 — Supplementary Information. [file 41598_2022_19005_MOESM1_ESM.pdf]

Supplementary material for "**Knowledge of perturbation design is essential for accurate gene regulatory network inference**"

Deniz Seçilmiş[1], Thomas Hillerton[1], Andreas Tjärnberg[2], Sven Nelander[3], Torbjörn E. M. Nordling[4,5], Erik L. L. Sonnhammer[1]*

[1]Department of Biochemistry and Biophysics, Stockholm University, Science for Life Laboratory, Box 1031, 17121 Solna, Sweden,
[2]Center for Developmental Genetics, New York University, New York, USA,
[3]Department of Immunology, Genetics and Pathology and Science for Life Laboratory, Uppsala University, SE-75185 Uppsala, Sweden
[4]Department of Mechanical Engineering, National Cheng Kung University, 701 Tainan, Taiwan
[5]Department of Applied Physics and Electronics, Umeå University, 90187 Umeå, Sweden
*To whom correspondence should be addressed: erik.sonnhammer@scilifelab.se

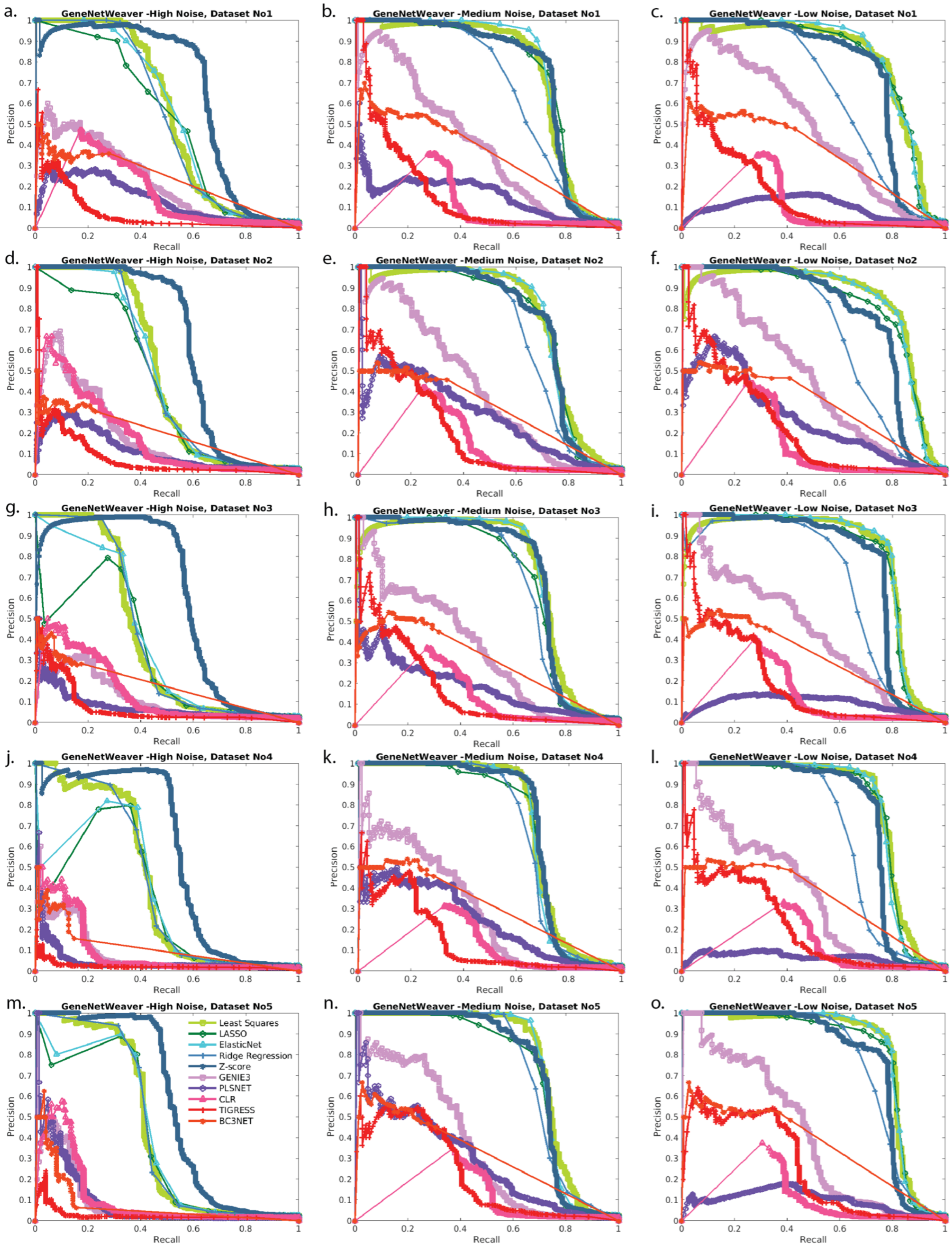

Figure S1. The precision-recall curves for data from the 100-gene GeneNetWeaver with **(a,d,g,j,m)** high, **(b,e,h,k,n)** medium, and **(c,f,i,l,o)** low noise levels for 5 datasets.

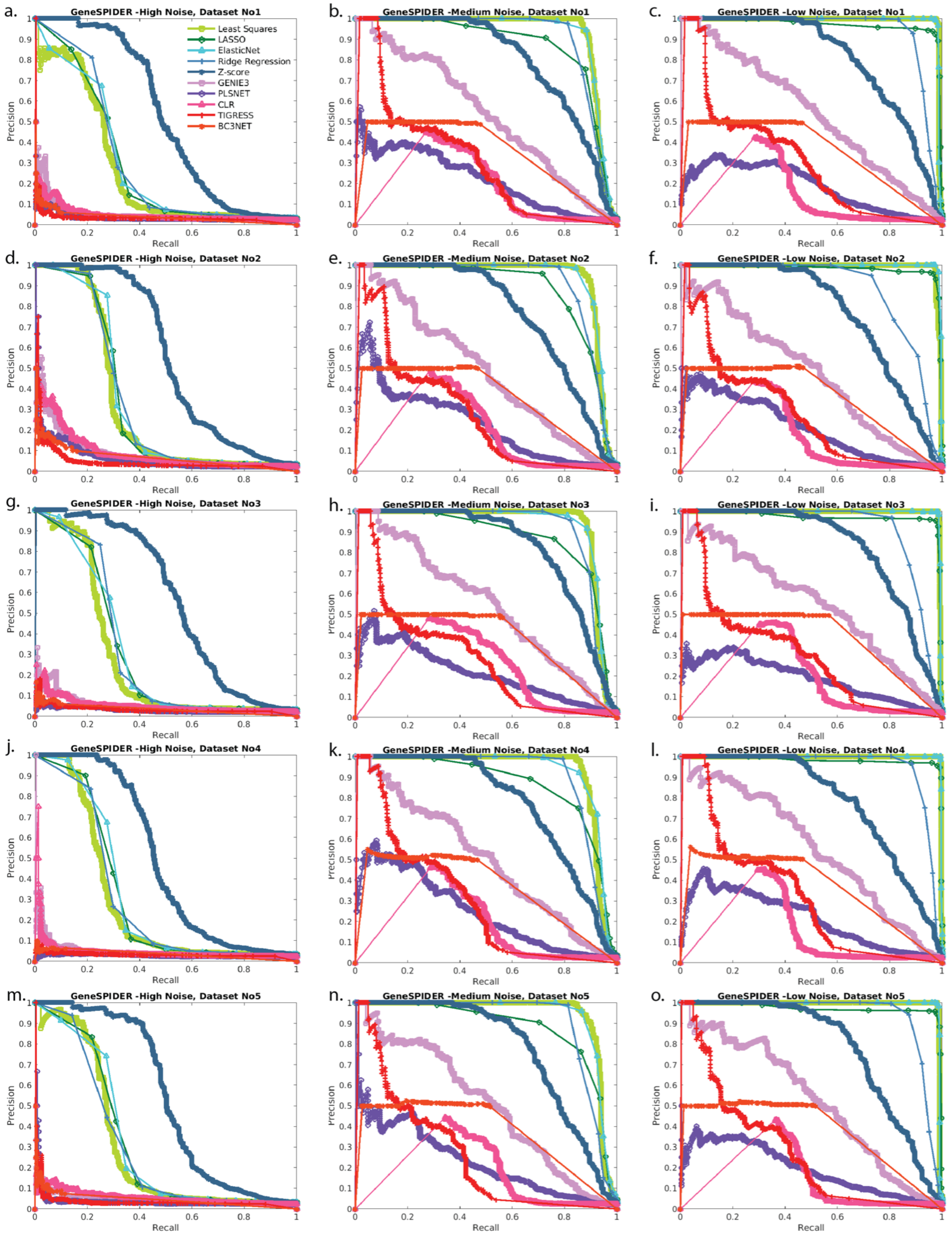

Figure S2. The precision-recall curves for data from the 100-gene GeneSPIDER with **(a,d,g,j,m)** high, **(b,e,h,k,n)** medium, and **(c,f,i,l,o)** low noise levels for 5 datasets.

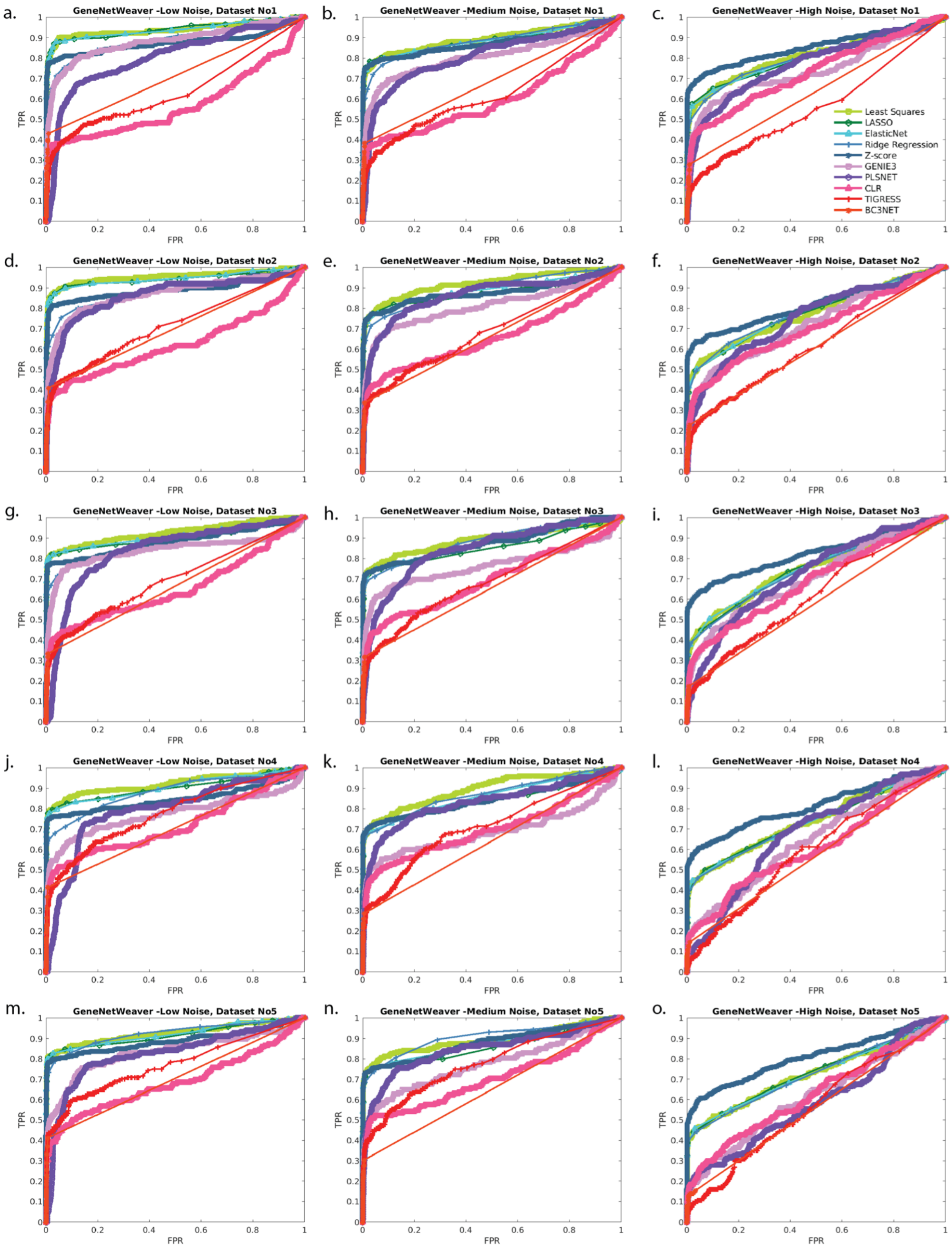

Figure S3. The receiver-operating-characteristic (ROC) curves for data from the 100-gene GeneNetWeaver with **(a,d,g,j,m)** high, **(b,e,h,k,n)** medium, and **(c,f,i,l,o)** low noise levels for 5 datasets.

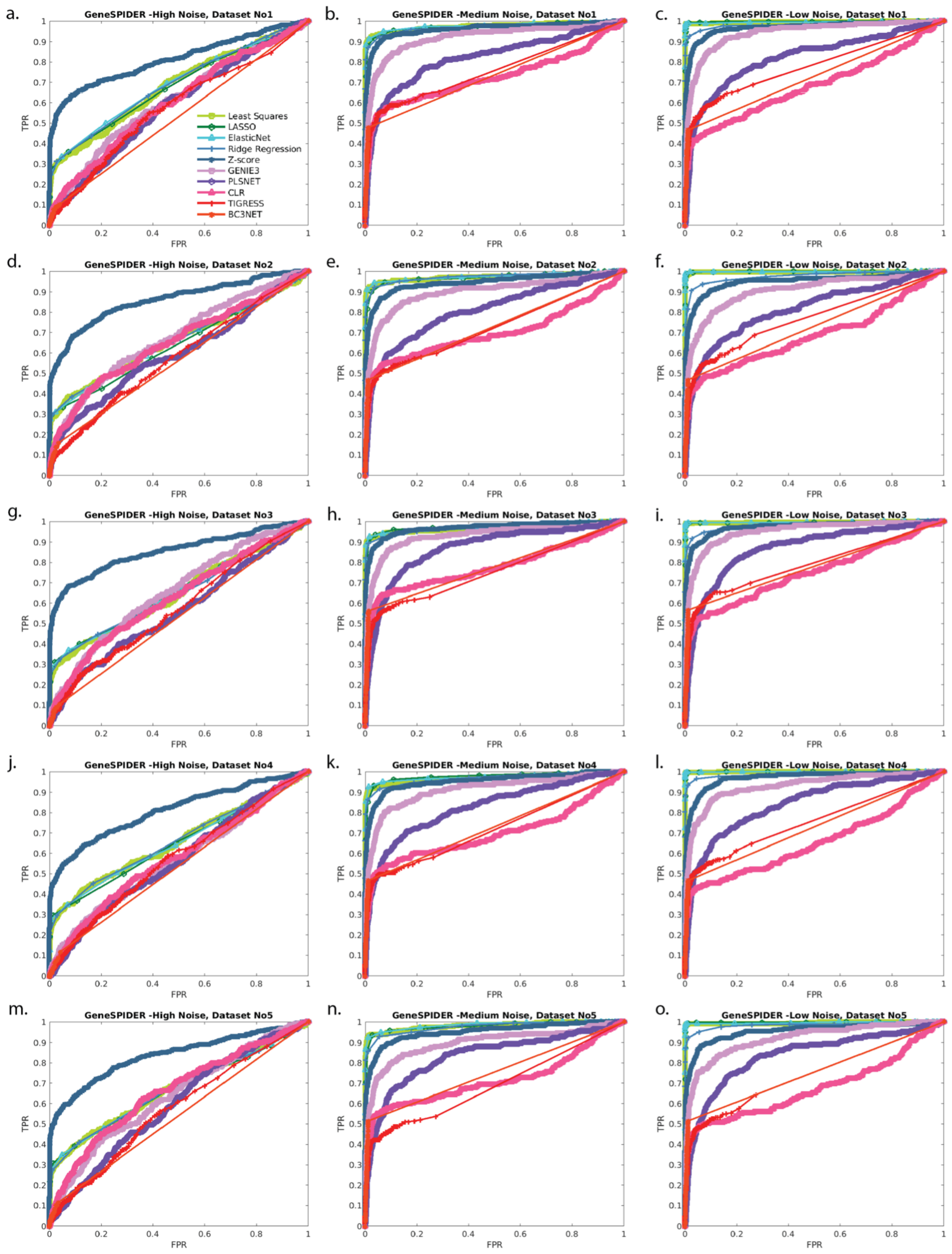

Figure S4. The receiver-operating-characteristic (ROC) curves for data from the 100-gene GeneSPIDER with **(a,d,g,j,m)** high, **(b,e,h,k,n)** medium, and **(c,f,i,l,o)** low noise levels for 5 datasets.

Table S1. Statistical significance from a two-tailed unpaired Mann-Whitney-U test of the differences between *P*-based and Non *P*-based methods, and between *P*-based methods provided with the correct and incorrect *P*-matrices, in terms of area under precision-recall (AUPR) curves. * denotes statistical significance with $p < 0.05$.

Data generation tool	Comparison	Noise Level	P-value
GeneNetWeaver (N100)	P-based vs Non P-based methods	High	0.00*
		Medium	0.00*
		Low	0.00*
	P-based vs incorrect P-based methods	High	0.00*
		Medium	0.00*
		Low	0.00*
GeneSPIDER (N100)	P-based vs Non P-based methods	High	0.00*
		Medium	0.00*
		Low	0.00*
	P-based vs incorrect P-based methods	High	0.00*
		Medium	0.00*
		Low	0.00*

Table S2. Statistical significance from a two-tailed unpaired Mann-Whitney-U test of the differences between the transitions from high to medium and from medium to low noise levels for both method categories. * denotes statistical significance with $p < 0.05$.

Data generation tool	Comparison	Perturbation Category	P-value
GeneNetWeaver (N100)	High vs Medium Noise	P-based methods	0.00*
	Medium vs Low Noise		0.00*
	High vs Medium Noise	Non P-based methods	0.00*
	Medium vs Low Noise		0.88
GeneSPIDER (N100)	High vs Medium Noise	P-based methods	0.00*
	Medium vs Low Noise		0.00*
	High vs Medium Noise	Non P-based methods	0.00*
	Medium vs Low Noise		0.82

Table S3. Statistical significance from a two-tailed unpaired Mann-Whitney-U test of the method similarity where 10 *P*-based method pairs were compared to 10 Non *P*-based method pairs at each noise level. * denotes statistical significance with $p < 0.05$.

	Noise		
**Data generation tool**	**High**	**Medium**	**Low**
GeneNetWeaver (N100)	1.299011e-04*	1.082509e-05*	1.082509e-05*
GeneSPIDER (N100)	1.082509e-05*	2.165018e-05*	1.082509e-05*

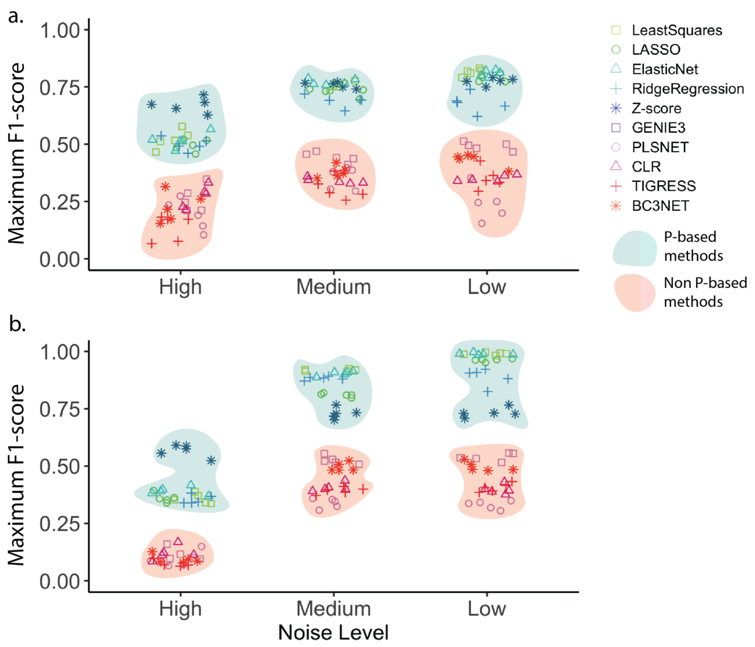

Figure S5. Accuracy of the GRN inference in terms of the F1-score from the 100-gene **(a)** GeneNetWeaver and **(b)** GeneSPIDER datasets. The x-axis represents different noise levels, and the y-axis denotes the maximum F1-scores calculated over different sparsities. Each method has five data points for each noise level for data generated from different true GRNs. The *P*-based and non *P*-based methods are represented by different markers and colors, and are highlighted together with blue and red, respectively.

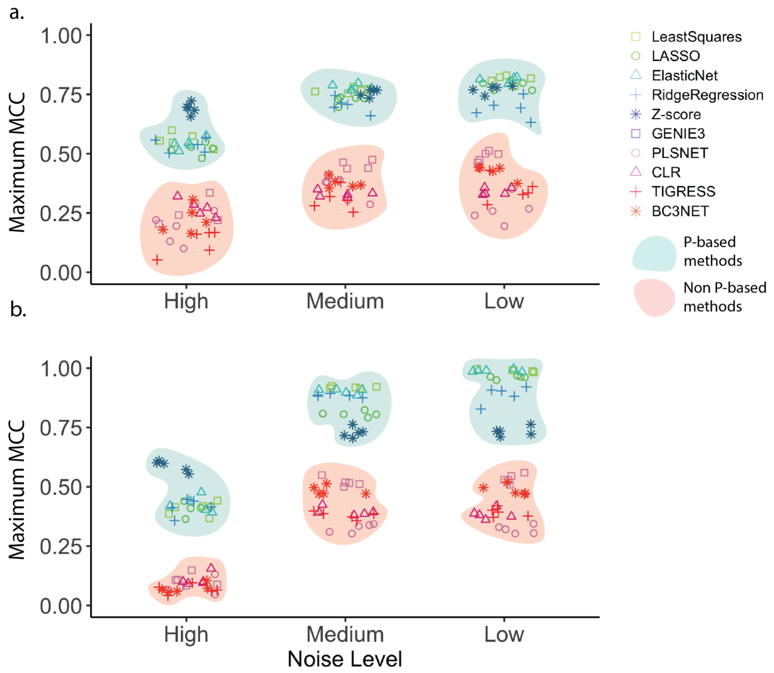

Figure S6. Accuracy of the GRN inference in terms of Matthew's correlation coefficient (MCC) from the 100-gene **(a)** GeneNetWeaver and **(b)** GeneSPIDER datasets. The x-axis represents different noise levels, and the y-axis denotes the maximum MCC levels calculated over different sparsities. Each method has five data points for each noise level for data generated from different true GRNs. The *P*-based and non *P*-based methods are represented by different markers and colors, and are highlighted together with blue and red, respectively.

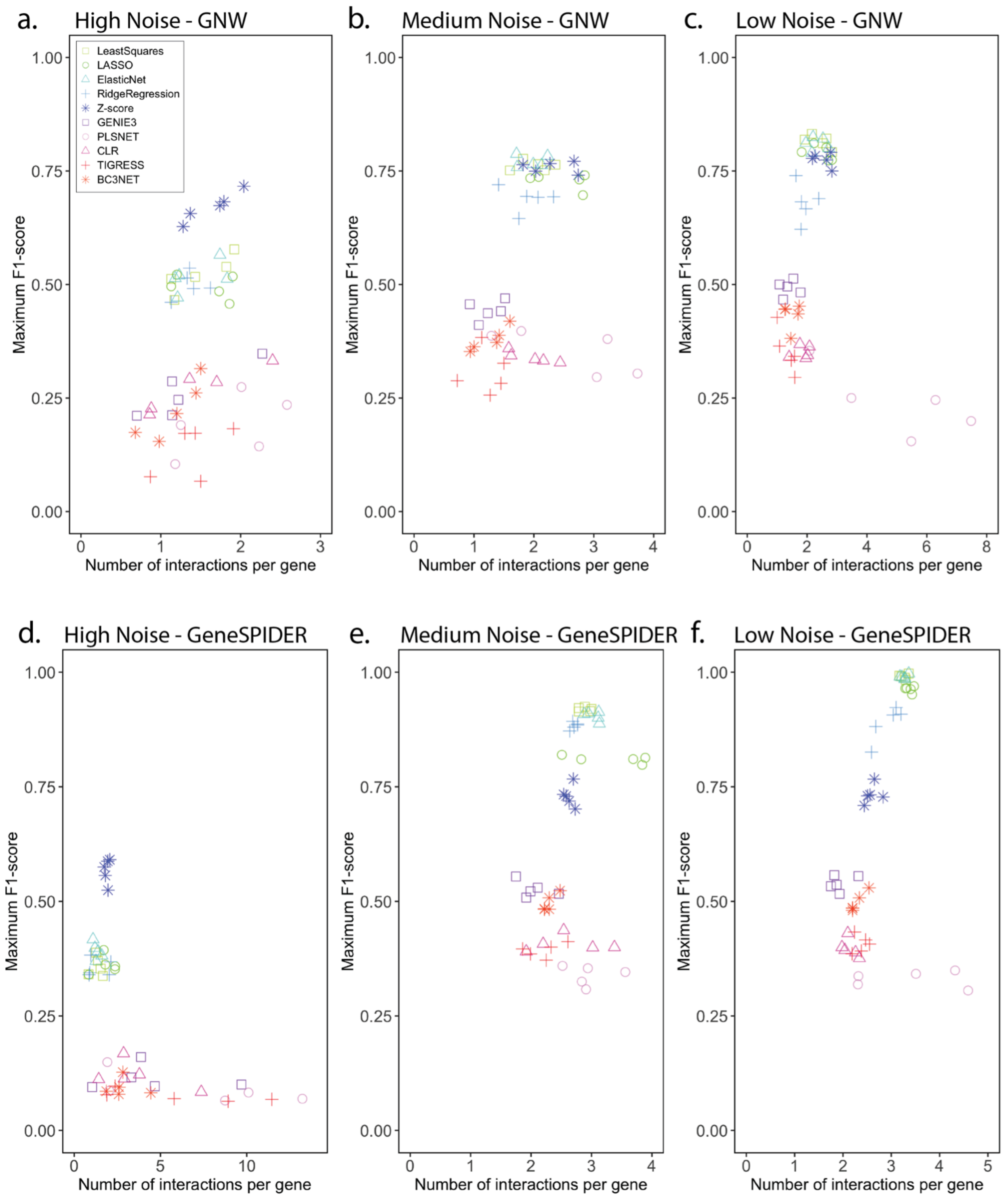

Figure S7. Sparsity of the GRNs with the maximum F1-scores **(a-c)** for the 100-gene GeneNetWeaver (GNW) data, and **(d-f)** for the 100-gene GeneSPIDER data at high noise (left panels), medium noise (middle panels) and low noise (right panels) levels.

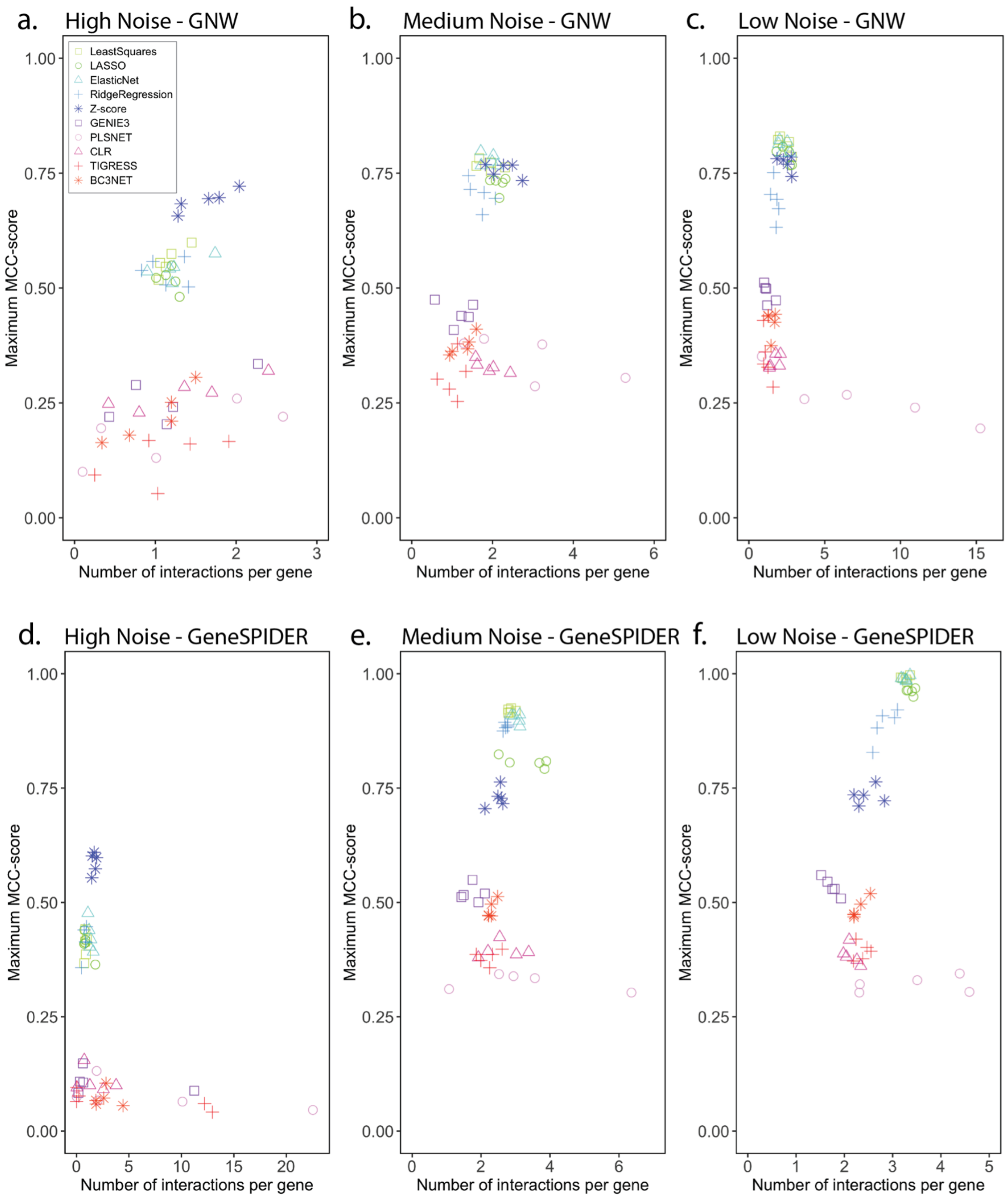

Figure S8. Sparsity of the GRNs with the maximum Matthew's correlation coefficient (MCC) levels **(a-c)** for the 100-gene GeneNetWeaver (GNW) data, and **(d-f)** for the 100-gene GeneSPIDER data at high noise (left panels), medium noise (middle panels) and low noise (right panels) levels.

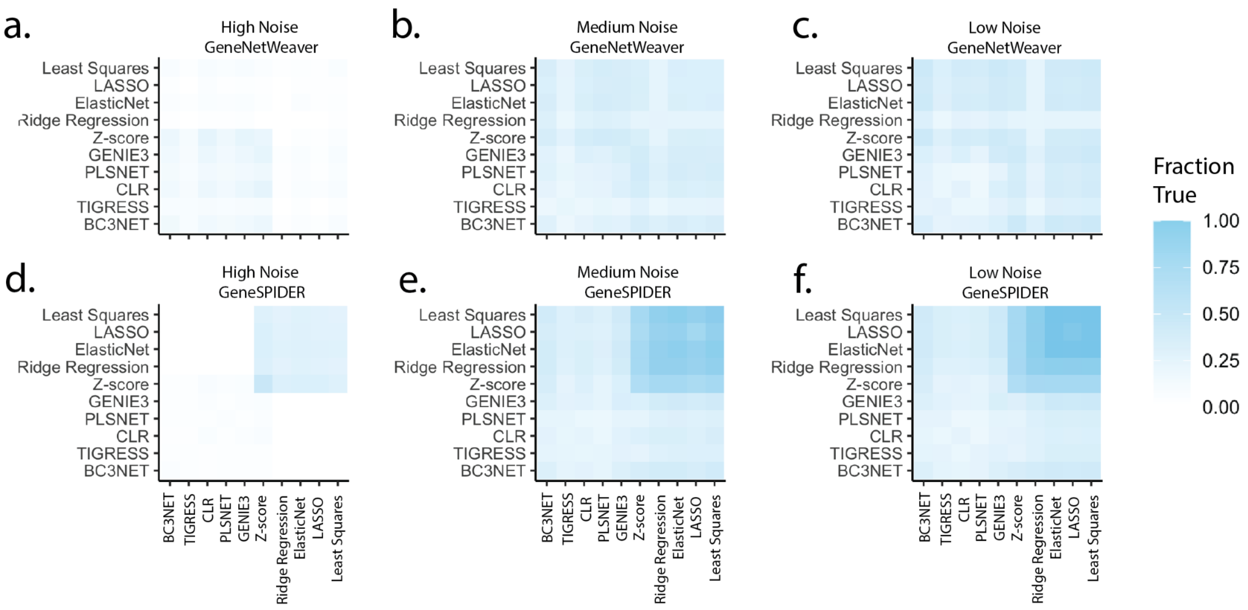

Figure S9. Average true link fraction in the overlap between the interactions predicted by the benchmarked methods across 5 100-gene datasets for each of the three noise levels, high (left column), medium (middle column), and low (right column) noise levels for GeneNetWeaver **(a-c)** and for GeneSPIDER **(d-f)** data.

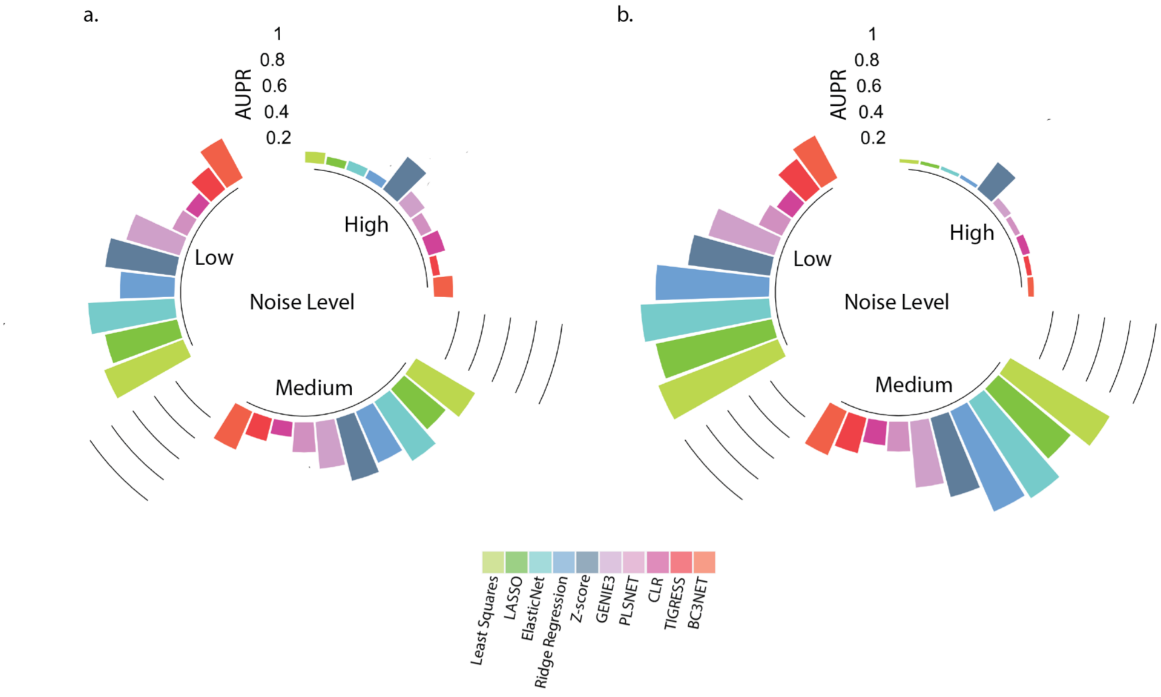

Figure S10. Accuracy of the GRN inference via the alternative benchmarking method where the self loops are removed from both the true and inferred GRNs of all methods in terms of the area under the precision-recall (AUPR) curve. Inference accuracy in terms of AUPR was averaged across 5 100-gene datasets from **(a)** GeneNetWeaver and **(b)** GeneSPIDER datasets. The circular x-axis represents different noise levels, and the y-axis denotes the AUPR levels calculated over different sparsities.

Table S4. Statistical significance from a two-tailed unpaired Mann-Whitney-U test of differences between the *P*-based methods when self loops are removed and Non *P*-based methods. * denotes statistical significance with $p < 0.05$.

Data generation tool	Comparison	Noise Level	P-value
GeneNetWeaver (N100)	P-based without self loops vs Non P-based	High	0.31
		Medium	0.00*
		Low	0.00*
GeneSPIDER (N100)		High	0.00*
		Medium	0.00*
		Low	0.00*

Table S5. The performances of the benchmarked methods on the 100-gene GeneNetWeaver - Dataset1.

Method	CPU Time (s)	Real Time (s)
Least Squares	2.43	1.65
LASSO	3.74	2.71
ElasticNet	2.36	2.23
Ridge Regression	7.74	1.69
Z-score	0.51	0.24
GENIE3	199.61	198.71
PLSNET	461.27	54.12
CLR	0.48	0.80
TIGRESS	2609.20	82.32
BC3NET	1.08	1.08

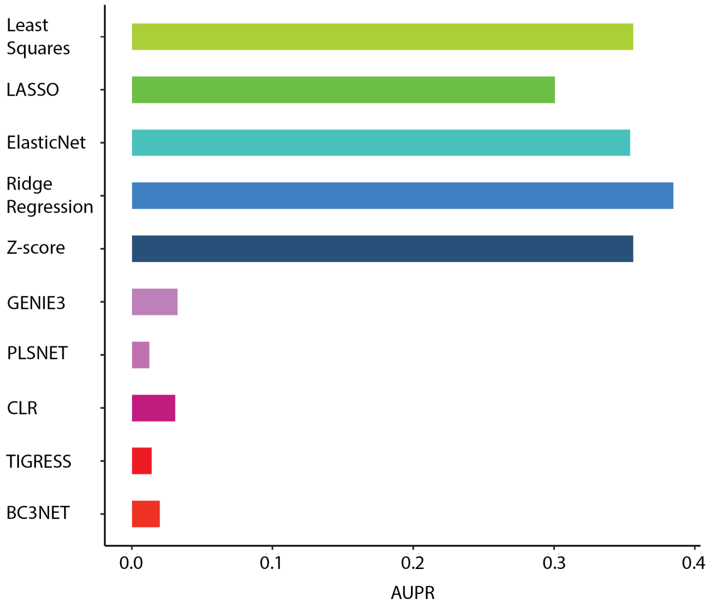

Figure S11. Accuracy in terms of area under the precision-recall (AUPR) curve from the DREAM5 *E. coli* (network3) subset data.

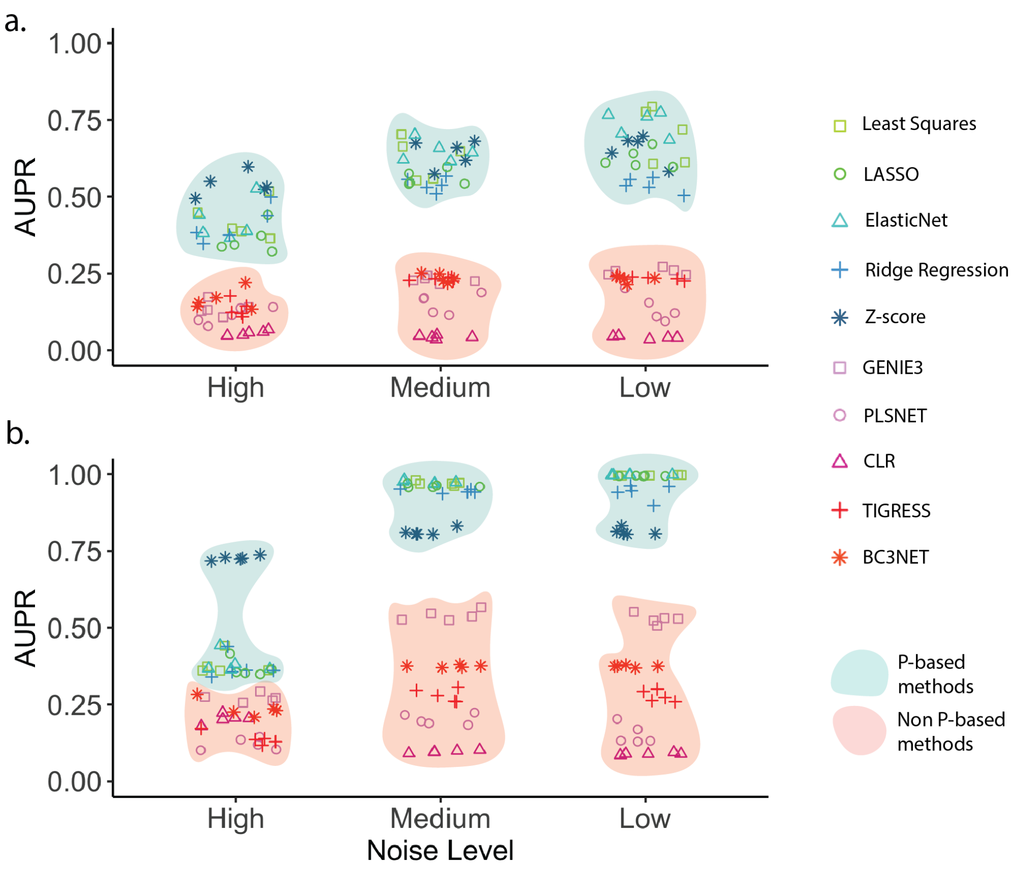

Figure S12. Accuracy of the GRN inference in terms of area under precision-recall (AUPR) from the 250-gene **(a)** GeneNetWeaver and **(b)** GeneSPIDER datasets. The x-axis represents different noise levels, and the y-axis denotes the maximum MCC levels calculated over different sparsities. Each method has five data points for each noise level for data generated from different true GRNs. The *P*-based and non *P*-based methods are represented by different markers and colors, and are highlighted together with blue and red, respectively.

Supplementary Note 1. Investigating the edge direction in GENIE3 GRNs and potential causes for the observed improvement when reversing the direction.

Before benchmarking, we tested all methods on their own published datasets, in most cases the *in silico* multifactorial data from the fourth round of DREAM network inference challenges to avoid potential bugs and/or uncertainties prior to GRN inference on our own generated data, and noticed a strange situation in GENIE3 GRNs. Even though we were able to reproduce the accuracy results from the DREAM4 *in silico* multifactorial challenge in terms of both AUROC and AUPR by using the original GENIE3 GRN format, the reverse edge direction greatly outperformed the original direction on our own 100-gene datasets generated via both GeneNetWeaver and GeneSPIDER. We investigated potential reasons for this situation, and observed that in most cases the reverse direction had a higher weight than the original one in the inferred GRNs, causing greatly improved accuracy when the output GRN matrix was transposed (Suppl. Fig. S14).

Bidirectional edges in GRNs

We investigated the fraction of bidirectional edges in both the true GRNs and the ones inferred by GENIE3. True GRNs from both GeneNetWeaver and GeneSPIDER have a sparse topology whereas GENIE3 infers a denser, fully connected, GRN consisting of weighted edges. A sparser GRN that has the exact number of interactions as the true GRN was selected, and the bidirectionality of the GRN was calculated by the fraction of bidirectional edges for which a nonzero value exists in both directions to the total number of edges in the GRN. As seen in Suppl. Fig. S13, GRNs inferred by Genie3 contain many more bidirectional links than the corresponding true GRN.

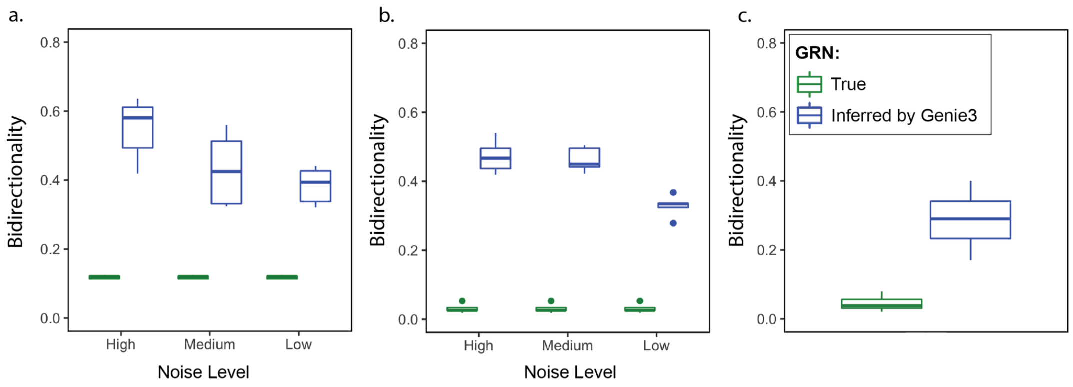

Figure S13. Fraction of bidirectional edges to the total number of edges in the GRN, in the true and inferred GRNs by GENIE3 using data from the 100-gene **(a)** GeneNetWeaver, **(b)** GeneSPIDER or **(c)** DREAM4. True and inferred GRNs have the same sparsity. The x-axis represents the noise level of the gene expression dataset from which GENIE3 inferred GRNs, and the y-axis denotes the bidirectionality of the GRN.

Fraction of regulators in GRNs

We investigated the fraction of regulators in both the true GRNs and the ones inferred by GENIE3. About half of the genes in the 100-gene GeneNetWeaver true GRNs are regulators, and about 85% in GeneSPIDER (Suppl. Fig. S14a and b). As seen in Suppl. Fig. S14c and d, there is a strong bias in the link direction for medium and low noise, favoring the reverse direction. This in turn results in higher accuracy for the reverse direction (Suppl. Fig. S14e and f) in terms of the area under precision-recall (AUPR).

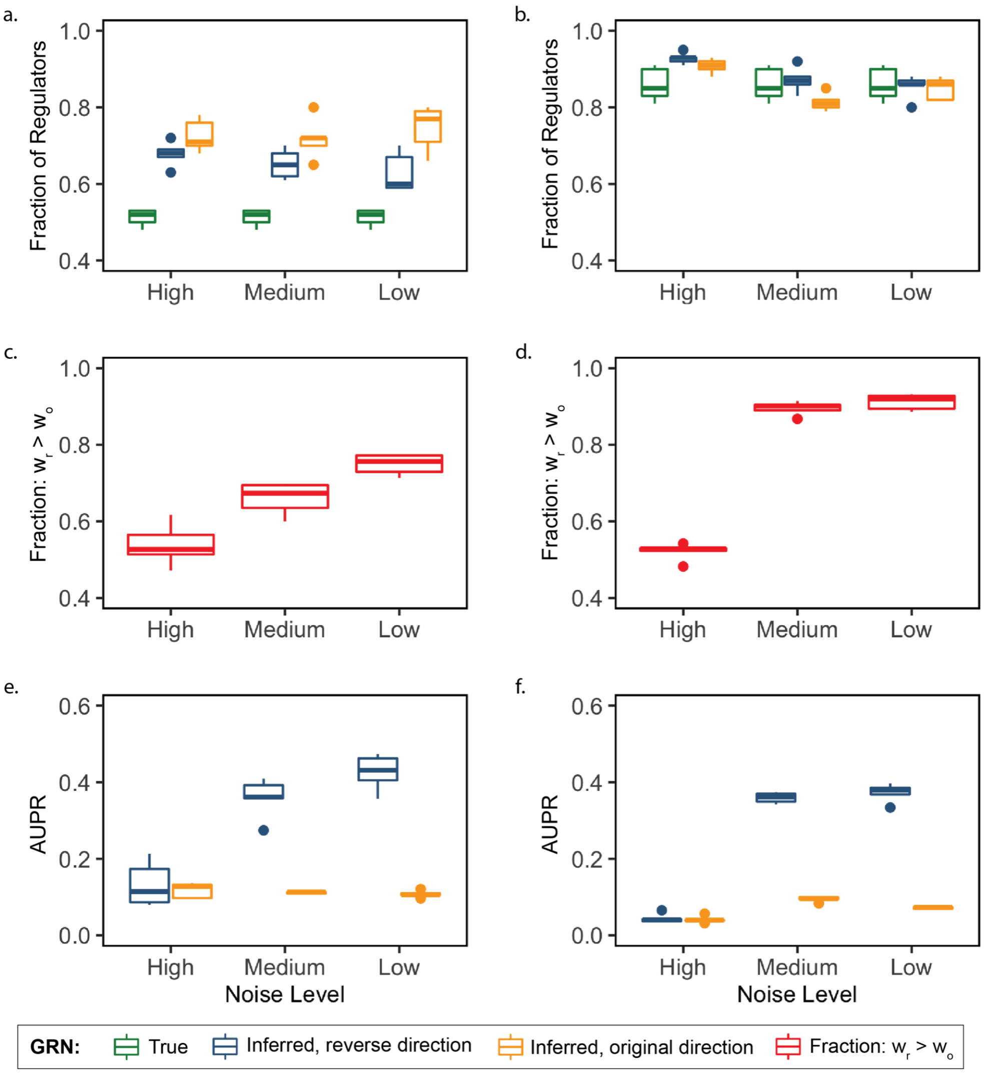

Figure S14. Fraction of regulators to the total number of genes in the true and inferred GRNs using the 100-gene data from **(a)** GeneNetWeaver and **(b)** GeneSPIDER. The fraction of all interactions inferred by GENIE3 (fully connected GRN) that overlap with the true GRN where the reverse edge direction has a higher weight than the original is shown for the 100-gene data from **(c)** GeneNetWeaver and **(d)** GeneSPIDER. w_r and w_o refer to the edge weight in the reverse and original directions, respectively. The accuracy of the inferred GRNs, both in the reverse edge direction and the original, is shown in terms of area under precision-recall (AUPR) for the 100-gene data from **(e)** GeneNetWeaver and **(f)** GeneSPIDER. The x-axis represents the noise level of the gene expression dataset from which GENIE3 inferred GRNs.

Analyses on the DREAM4 *in silico* multifactorial network inference challenge data

The analyses performed for the GeneNetWeaver and GeneSPIDER data were repeated on the data from DREAM4 (Suppl. Fig. S15). In this data, the difference in accuracy for the two directions is very small, hence the link directionality in the inferred GRN is of little consequence for this particular dataset.

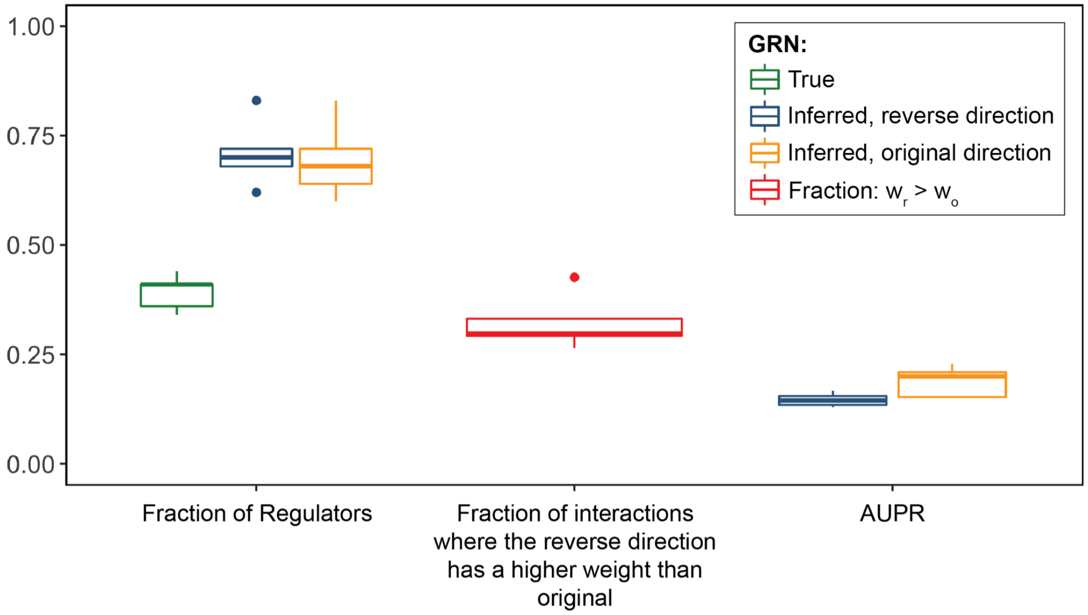

Figure S15. Fraction of regulators and link direction effect on GRN inference accuracy in GENIE3 on the data from DREAM4. The fraction of regulators to the total number of genes in the true and inferred GRNs at the true sparsity, the fraction of all interactions inferred by GENIE3 (fully connected GRN) that overlap with the true GRN where the reverse edge direction has a higher weight than the original (w_r and w_o refer to the edge weight in the reverse and original directions, respectively), and the accuracy of the inferred GRNs, both in the reverse edge direction and the original, are placed on the x-axis, and their values are shown on the y-axis. The GRNs were inferred by GENIE3 from the five DREAM4 *in silico* multifactorial datasets.
